# Unveiling lignocellulolytic potential: a genomic exploration of bacterial lineages within the termite gut

**DOI:** 10.1186/s40168-024-01917-7

**Published:** 2024-10-15

**Authors:** João Felipe M. Salgado, Vincent Hervé, Manuel A. G. Vera, Gaku Tokuda, Andreas Brune

**Affiliations:** 1 RG Insect Microbiology and Symbiosis, Max Planck Institute for Terrestrial Microbiology, 35043 Marburg, Germany; 2 Tropical Biosphere Research Center, Center of Molecular Biosciences, University of the Ryukyus, Nishihara, Okinawa 903-0213 Japan

**Keywords:** CAZymes, Lignocellulose degradation, Lignin, Functional genomics, Cellulase, Termite microbiota

## Abstract

**Background:**

The microbial landscape within termite guts varies across termite families. The gut microbiota of lower termites (LT) is dominated by cellulolytic flagellates that sequester wood particles in their digestive vacuoles, whereas in the flagellate-free higher termites (HT), cellulolytic activity has been attributed to fiber-associated bacteria. However, little is known about the role of individual lineages in fiber digestion, particularly in LT.

**Results:**

We investigated the lignocellulolytic potential of 2223 metagenome-assembled genomes (MAGs) recovered from the gut metagenomes of 51 termite species. In the flagellate-dependent LT, cellulolytic enzymes are restricted to MAGs of *Bacteroidota* (*Dysgonomonadaceae*, *Tannerellaceae*, *Bacteroidaceae*, *Azobacteroidaceae*) and *Spirochaetota* (*Breznakiellaceae*) and reflect a specialization on cellodextrins, whereas their hemicellulolytic arsenal features activities on xylans and diverse heteropolymers. By contrast, the MAGs derived from flagellate-free HT possess a comprehensive arsenal of exo- and endoglucanases that resembles that of termite gut flagellates, underlining that *Fibrobacterota* and *Spirochaetota* occupy the cellulolytic niche that became vacant after the loss of the flagellates. Furthermore, we detected directly or indirectly oxygen-dependent enzymes that oxidize cellulose or modify lignin in MAGs of *Pseudomonadota* (*Burkholderiales*, *Pseudomonadales*) and *Actinomycetota* (*Actinomycetales*, *Mycobacteriales*), representing lineages located at the hindgut wall.

**Conclusions:**

The results of this study refine our concept of symbiotic digestion of lignocellulose in termite guts, emphasizing the differential roles of specific bacterial lineages in both flagellate-dependent and flagellate-independent breakdown of cellulose and hemicelluloses, as well as a so far unappreciated role of oxygen in the depolymerization of plant fiber and lignin in the microoxic periphery during gut passage in HT.

Video Abstract

**Supplementary Information:**

The online version contains supplementary material available at 10.1186/s40168-024-01917-7.

## Background

Termites are eusocial insects that effectively digest wood and other lignocellulosic plant matter [1]. Their degradation mechanism involves a dual system. The termite comminutes the wood particles and degrades amorphous regions of the cellulose with endoglucanases and β-glucosidases secreted in the salivary glands or the midgut [2, 3], and symbiotic microorganisms in the dilated hindgut subsequently degrade the wood particles that pass from the midgut into the hindgut largely intact [4]. The contributions of microbial symbionts to fiber digestion are essential as the host lacks both exoglucanases acting on crystalline cellulose and any hemicellulolytic activities.

The microbial community in the hindgut of termites is highly specialized and differs between lineages [5]. Flagellates dominate the microbiota of most termite families (collectively referred to as lower termites, LT), where they sequester wood particles from the hindgut fluid into digestive vacuoles containing both endoglucanases and exoglucanases [55], suggesting that bacteria are of little relevance in the digestion of plant fiber for these termites [5]. In the family Termitidae (higher termites, HT), the loss of cellulolytic flagellates is compensated by the cellulolytic activity of their bacterial microbiota [6, 7]. In the subfamily Macrotermitinae, which cultivate basidiomycete fungi of the genus *Termitomyces* that degrade (hemi)cellulose and depolymerize lignin [8], the need for degradation during gut passage is alleviated by the pretreatment of lignocellulose in fungal gardens [9].

Therefore, the breakdown of recalcitrant fibers in the hindgut of most HT is an entirely bacterial process [2]. Metagenomic studies revealed the presence of carbohydrate-active enzymes (CAZymes) that are often closely related to those of cellulolytic clades from the rumen of cattle or other environments [10–12]. However, these studies covered only a few species of higher termites, and from the arsenal of glycoside hydrolases (GHs), only a few cellulases (GH5) of unidentified hindgut bacteria and hemicellulases (GH10 and GH11) of *Treponematales* have been characterized [12–14].

Apart from a recent study, which analyzed the CAZymes in a wide range of metagenomes from many termite families and documented considerable differences in the CAZyme repertoire of LT and HT [15], little is known about the fiber-digesting potential of specific bacterial lineages of the gut microbiota in LT. Most importantly, only few of the CAZymes identified in the metagenomic datasets have been pinpointed to specific bacterial lineages using metagenome-assembled genomes (MAGs) [10, 16, 18]. Moreover, most previous analyses focused on GHs but did not discuss other CAZyme families acting on lignocellulose, such as carbohydrate-binding modules (CBM) and auxiliary activities (AA). CBMs are enzyme domains involved in the attachment of GHs to their respective substrate, whereas some AAs participate in redox reactions that depolymerize (hemi)celluloses and lignin and are directly or indirectly dependent on molecular oxygen as co-substrate [17]. Certain carbohydrate esterases (CE) remove methyl and acetyl groups from the polysaccharide backbone of hemicelluloses and, together with polysaccharide lyases (PL), also contribute to pectin degradation. Glycosyl transferases (GT) are mostly involved in the biosynthesis of glycoproteins and glycolipids [18–20].

Here, we present a comprehensive genome-centric analysis of the CAZymes encoded by more than 2000 bacterial MAGs reconstructed from gut metagenomes of 51 termite species [21, 22]. Using a vast genomic library, covering nearly all termite families, we identify the distribution of CAZymes among all major bacterial lineages previously detected in termite guts. We focus on GHs, AAs, and CBMs with lignocellulolytic activity among MAGs from individual bacterial lineages from different groups of termites, for the first time including also oxygen-dependent activities.

## Methods

### Metagenome-assembled genomes and phylogenomics

MAGs were generated in previous metagenomic studies from our group [21, 22]. Metadata associated with the corresponding metagenomes can be found in Supplementary Table S1. The quality of the reconstructed genomes was estimated with CheckM v1.1.6 [23]. MAGs that were at least 50% complete and less than 10% contaminated were retained for analyses. Relative abundances were calculated as previously described [22]. MAGs were classified using the Genome Taxonomy Database (GTDB) with the GTDB-Tk v2 toolkit [24], based on an alignment of 120 ubiquitous marker genes retrieved from the MAGs, and phylogenies were reconstructed using IQ-TREE [25]. To validate genome classifications, taxonomies from Pplacer v1.1.17 [26] and CheckM were taken into consideration. Metadata associated with each MAG can be found in Supplementary Table S2. The termite phylogeny is based on previous work [15, 27, 28], using a combination of rRNA genes and mitochondrial genomes. All maximum-likelihood phylogenetic trees were curated using the packages ape [29] and PAML [30]. Genomic data were mapped to phylogenies using iTOL [31] and R package *ggtree* [32].

### Gene prediction and annotation

Genomes were subjected to gene prediction using Prodigal [33], with 4,480,144 genes predicted. Coding densities were also estimated by the software, by averaging the total CDS bases as a function of the total genome bases and expressed in percentages, and can be found in Table S2. Protein domain annotations were added using the TIGRFAM [34], Pfam [35], SUPERFAMILY [36], Panther [37], and CDD [38] databases in the INTERPROSCAN [39] classifier, with default parameters. CAZymes were identified using Hidden Markov Model (HMM) searches (e-value < 1e-15, score > 100, and coverage > 0.35) against the dbCAN3 and dbCAN-sub metaservers [40], using the function *run_dbcan*. The classification was confirmed with protein domain annotations and DIAMOND [41] searches (e-value < 1e-15, id > 50%) against the CAZy database [42]. Signal peptides were identified with SignalP V.6 [43] and Phobius [44].

### Multivariate analyses and cluster visualization

A dataset including all high-quality CAZyme genes with secretion signals was subjected to uniform manifold approximation and projection (UMAP), using the R package *umap* [45]. The k-nearest neighbors (KNN) algorithm was used with parameters for 15 neighbors and 500 epochs for training. For the input, the raw-count data were square-root transformed and Wisconsin double standardized with the function *decostand*, and a Bray–Curtis dissimilarity matrix was calculated using the function *vegdist*; both functions are from the R package *vegan* [46]*.* The analysis included host family and subfamily, termite diet, gut compartment, and bacterial phylum as metadata. The contribution of these variables to CAZyme composition was further estimated with the functions ADONIS and ANOSIM, also based on Bray–Curtis dissimilarities, each with 1000 permutations and bootstrapped 100 × for best model fit (max stress = 0.12) in the *vegan* package. Principal component analyses (PCA) were used as implemented in the function *prcomp* of the R package *stats* [47]. Explanatory PCs were employed in rarefactions for the relationship between CAZyme family distributions in relation to either the per-MAG number of genes predicted or metagenomic relative abundance.

### CAZyme family selection and statistical analyses

Outliers were removed, and homogeneity of variance and normality was checked using Levene’s and Shapiro Test from the R packages *car* [48] and *stats*. Since neither assumption was applicable, a nonparametric Kruskal–Wallis test was used on the entire pre-selection CAZyme dataset to determine significantly different CAZyme families across the 15 bacterial phyla, with the function *kruskal.test* and with post hoc Mann–Whitney *U* tests in the function *wilcox_test* of the package *rstatix* [49], followed by Bonferroni multiple testing correction at a threshold of *p* < 0.0001, using the *p.adjust* function of the *stats* package. A total of 146 CAZyme families tested positive for association with the phyla, of which those with lignocellulolytic activities (38 CAZyme families containing 8 CBMs) were selected for downstream comparison.

The classification of individual CAZymes into the major lignocellulolytic categories (cellulase, hemicellulase, ligninase, chitinase, and pectinase) was based on the gene-wise presence and complementary activities using the substrate predictions of DBCAN-sub (see Table S3). Enzymes with only endo-β-1,4-glucanase activities or other classically cellulolytic activities, such as cellobiohydrolases, were classified as cellulases, whereas enzymes with endo-β-1,4-glucanase activity and additional xylanolytic or xyloglucan-specific activities were classified as hemicellulases. Enzymes from a given CAZyme family with hemicellulolytic activities were considered hemicellulases even if they comprised endo-β-1,4-glucanase activity. Enzymes from family GH3 were not included in this categorization given the complexity of their predicted substrates. CBMs that were not connected to CAZymes assigned to specific GH families were removed from the dataset. CAZymes from families with peroxidase, aryl alcohol oxidase, and/or laccase activities were classified as ligninases. Pectate lyases and pectin methylesterases were classified as pectinases. Enzymes with acetylglucosaminosidase, chitin deacetylase, and chitinase activities were classified as chitinases.

To confirm bacterial phylogeny as a covariate, we employed a local indicator of phylogenetic association (lipaMoran) analysis coupled with an Abouheif test. CAZyme families containing (hemi)cellulases and lignin-modification enzymes were used as traits, and the phylogenetic signal was considered at *p*-values < 0.05. Correlograms were plotted with the R package *phylosignal* [50]. Since there was no considerable coding density variation between MAGs, gene densities (GD) were estimated by normalizing the numbers of genes in the lignocellulolytic CAZyme classifications by per-MAG number of predicted genes. Specific Wilcoxon’s signed-rank tests were used to assess the association of GD in HT and LT, and the effect sizes were estimated using the function *wilcox_effsize* of the package *rstatix*. Correlation between continuous variables was tested with Spearman correlation rank, as implemented by the function *cor.test* in the R package *stats*.

## Results

### Representation of bacterial MAGs and *phyla* in termite microbiomes

**Fig. 1:**
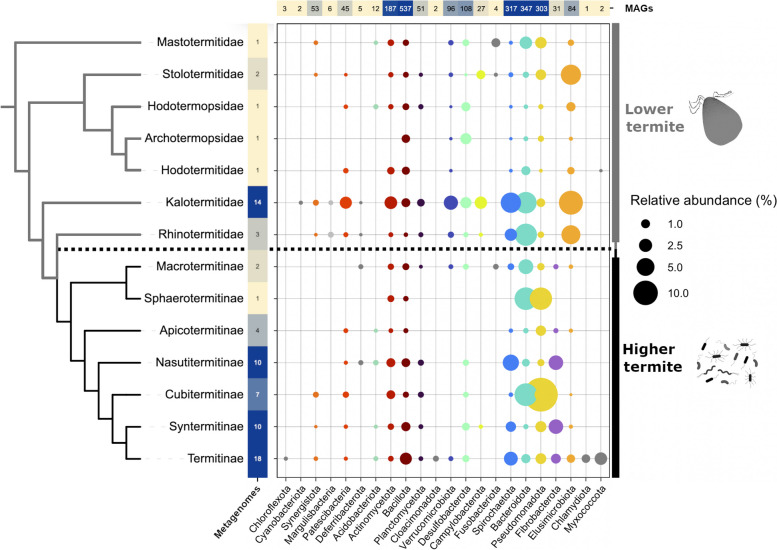
Distribution of MAGs from major bacterial phyla among metagenomes from different host groups. The ordinate shows the number of MAGs in each phylum as a heatmap that is based on the total number of MAGs (2,223) in the dataset. The abscissa shows the number of metagenomes from which the MAGs were recovered, summarized for each host group, as a heatmap based on the total number of metagenomes (51). Relative abundance values indicate the proportion of reads in a metagenome that mapped against the corresponding MAGs, expressed as averages per termite family (lower termites) or subfamily (higher termites, Termitidae). The termite species contained in each host group, the relative abundances of individual MAGs, and their classification down to genus level are given in Tables S1 and S2

The initial dataset comprised 2223 bacterial MAGs that were recovered from 51 termite species, spanning seven families of lower termites (LT) and seven subfamilies of higher termites (HT) (Fig. 1). Archaeal MAGs were excluded because preliminary analyses revealed negligible numbers of CAZymes. The MAGs represented 22 bacterial phyla, with the majority classified among *Bacillota* (24.1%), *Bacteroidota* (15.6%), *Spirochaetota* (14.2%), and *Pseudomonadota* (13.4%).

The MAGs from different bacterial phyla differed strongly in relative abundance, ranging between 0.004 and 19.8% (average 0.31%) of the reads in the corresponding metagenomes (Fig. 1). Certain phyla (e.g., *Elusimicrobiota* and *Verrucomicrobiota*) were more prevalent among lower termites (LT), whereas others were more prevalent (*Pseudomonadota*) in or even restricted to (*Fibrobacterota*) higher termites (HT). Many phyla were represented only in certain (sub)families of LT and HT but virtually absent in others (e.g., *Actinobacteriota*, *Bacteroidota*, *Desulfobacterota*, *Spirochaetota*, and *Patescibacteria*). The bacterial phyla *Chlamydiota*,* Cyanobacteriota*, *Myxococcota*, *Cloacimonadota*, Chloroflexota, *Fusobacteriota*, and *Deferribacteria* presented less than 5 MAGs and were removed from subsequent analyses.

**Fig. 2:**
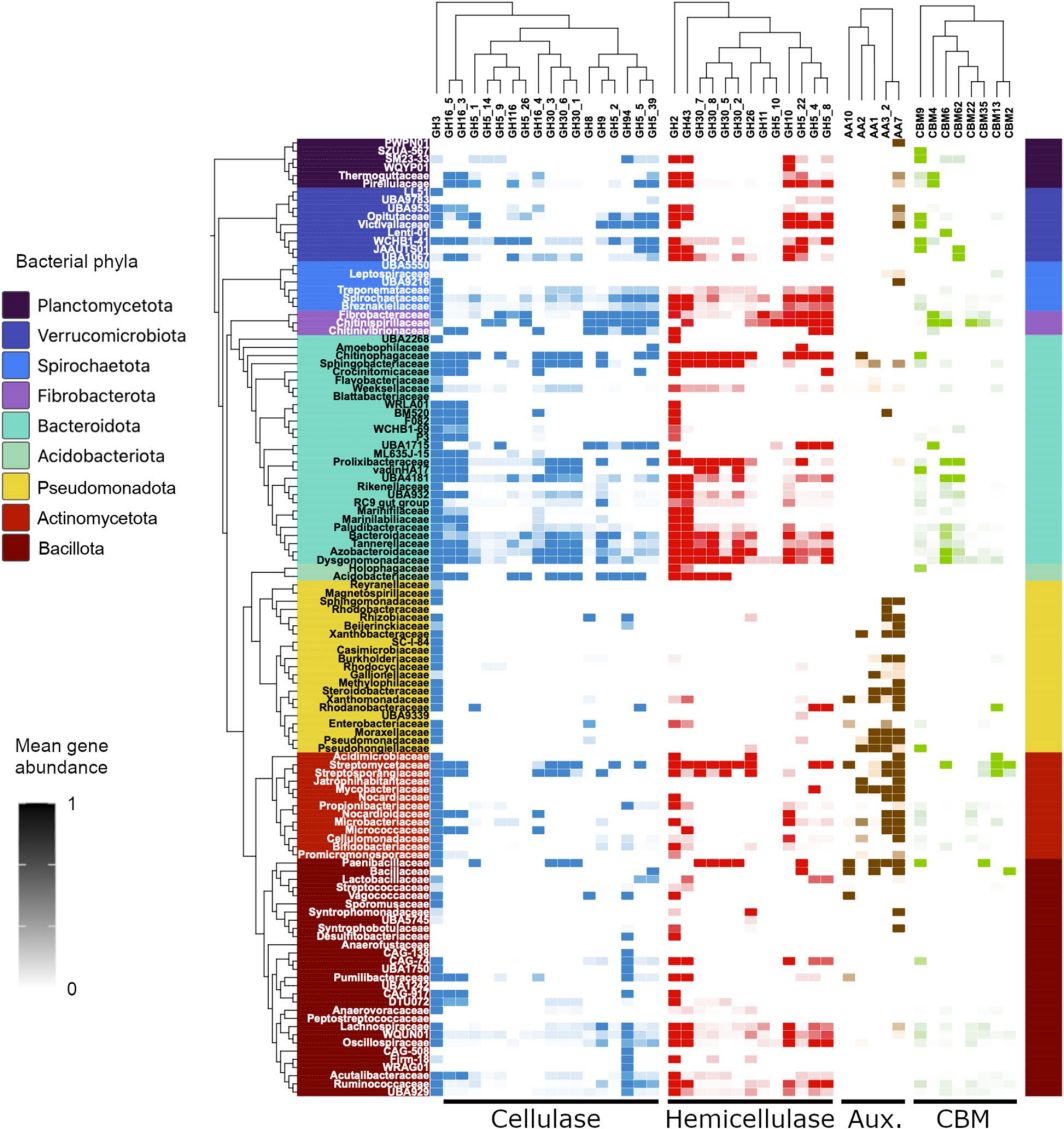
The distribution of carbohydrate-active enzymes with lignocellulolytic activities (cellulase, hemicellulase, auxiliary) and cellulose-binding modules (CBM) among the MAGs of major bacterial families recovered from termite gut metagenomes. The heatmap indicates the mean gene abundance in selected CAZyme families for the MAGs from the respective bacterial family. CAZYme families were clustered using a hierarchical clustering algorithm; values were scaled from 0 to 1 to facilitate visualization. An interactive spreadsheet with details for all MAGs is given in Table S4; the complete dataset is given in Table S5

The remaining 2204 MAGs from 15 bacterial phyla were analyzed for CAZyme distribution. From the full dataset (Table S5), we selected 38 CAZyme families with predicted secretion signals that are involved in lignocellulose degradation (Table S3) and were differentially distributed across phyla (*p* ≤ 0.001) (Fig. 2). They comprised 18 families with cellulolytic, 13 families with hemicellulolytic, and 7 families with lignin-modifying activities (Table S3), possessing additionally 8 CBM domain families. CAZyme families that are not directly involved in lignocellulolytic processes, such as glycosyltransferases (GT) and polysaccharide lyases (PL), were excluded from the analysis. Carbohydrate esterases (CE) of subfamily CE4 were represented in almost all MAGs (Table S5), but since their predicted substrates did not include acetylxylan, they were also excluded.

There were clear patterns in the distribution of the selected CAZymes among bacterial lineages (Fig. 2). Both cellulolytic and hemicellulolytic activities were prominent among the MAGs of *Bacteroidota*, especially *Sphingobacteriaceae*, *Chitinophagaceae*, and most families of *Bacteroidales*. MAGs of the *Fibrobacterota* possessed a high abundance of cellulolytic enzymes, with specific sets of CAZyme families that were not observed in other phyla. Large numbers of (hemi)cellulases were present also in MAGs of the *Bacillota* and *Actinobacteriota*. Among *Bacillota*, MAGs with considerable numbers of cellulases were found mostly among *Clostridia* (e.g., *Lachnospiraceae*, *Acutalibacteraceae*, and *Clostridiceae*), whereas the only prominent family with cellulases among *Bacilli* was *Paenibacillaceae*. Clear differences were apparent also in the distribution of auxiliary activities and CBMs (see below).

### Factors driving bacterial CAZyme distribution

**Fig. 3:**
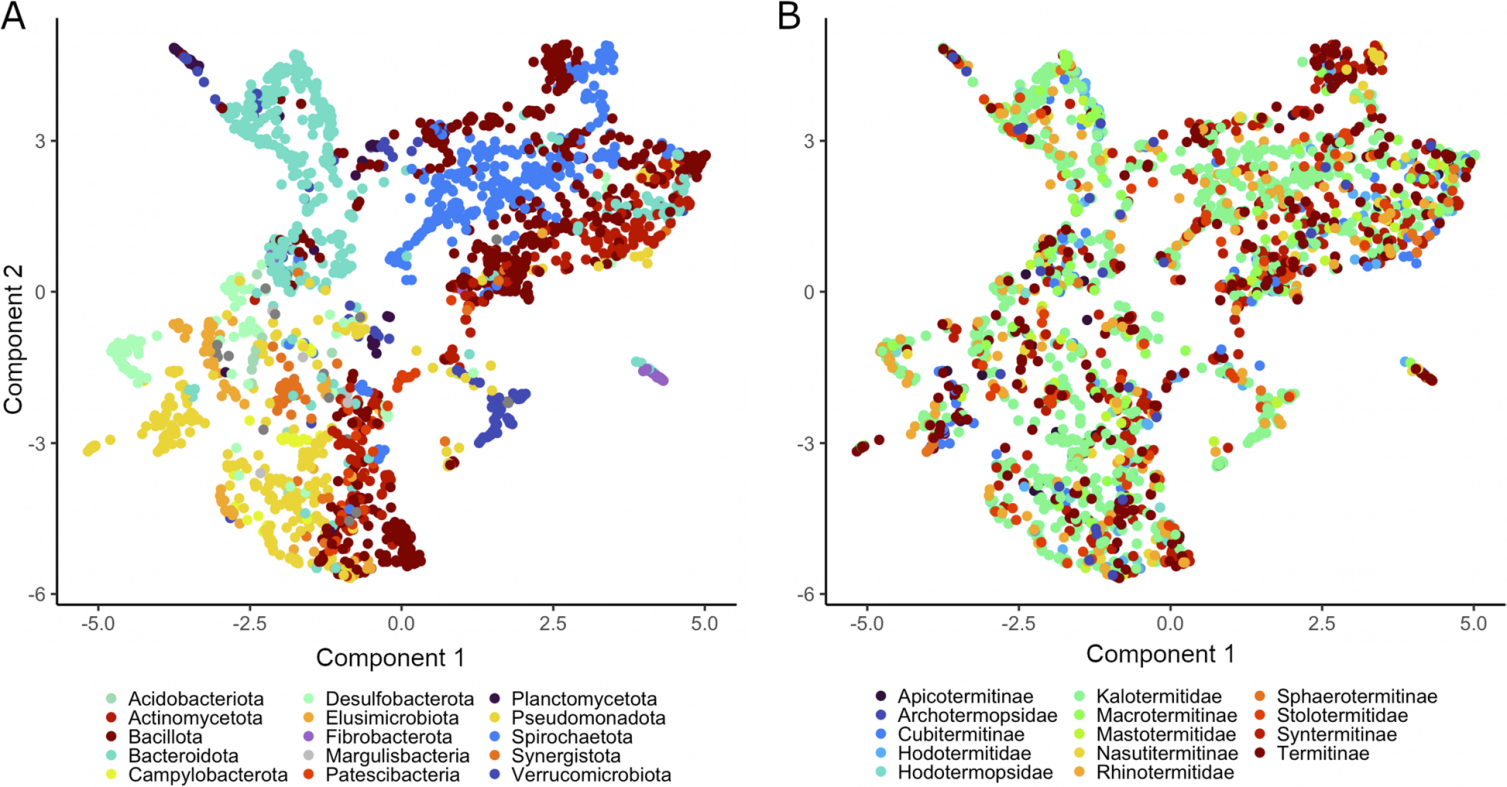
Ordination analysis of CAZyme composition in 2,204 MAGs of termite-associated bacteria. The UMAP analysis (stress = 0.12) used the entire dataset of CAZymes (256,212 genes across 534 CAZyme families; see Table S5) to visualize differences in the general content of CAZymes among phyla. The biplot displays MAGs color-coded according to their respective phyla (**A**) and according to the termite (sub)families from which they were recovered (**B**). The theoretical distances were calculated based on the KNN score, and the statistics are based on ANOSIM (bacterial phyla *R* = 0.34; termite (sub)families *R* = 0.04; *p* < 0.001) and ADONIS (bacterial phyla *R*^2^ = 0.24; termite (sub)families *R*^2^ = 0.03; *p* < 0.001) tests

Based on the obvious patterns in CAZyme distribution among MAGs from different bacterial lineages, we went back to the entire pre-selection dataset of CAZymes (256,212 genes across 534 CAZyme families; Table S5) to test whether the distribution patterns of CAZymes in the termite gut reflect bacterial taxonomy or host factors. Dimensionality reduction using all CAZyme families revealed that the bacterial phylum is an almost exclusive driver of CAZyme distribution (Fig. 3A), followed to a lesser extent by the (sub)family of its termite host (Fig. 3B). A separation of the MAGs from LT and HT reveals similar distribution patterns (Fig. S1A-B). Other factors, such as host diet or gut compartment, did not affect the ordination (Fig. S1C-D). A fitted phylogenetic signal model confirmed the association of bacterial taxonomy to the 38 selected CAZyme families. There was phylogenetic signal in distribution of cellulases in the phyla *Bacillota*, *Actinomycetota*, *Bacteroidota*, *Fibrobacterota*, and *Planctomycetota* (Figs. S2 and S3). The phylum *Spirochaetota* presented a phylogenetic signal for the distribution of hemicellulases. *Actinomycetota*, *Pseudomonadota*, and *Acidobacteriota* were the only phyla with a significant phylogenetic signal for the ligninases.

### Division of fibrolytic niches among bacterial clades

**Fig. 4:**
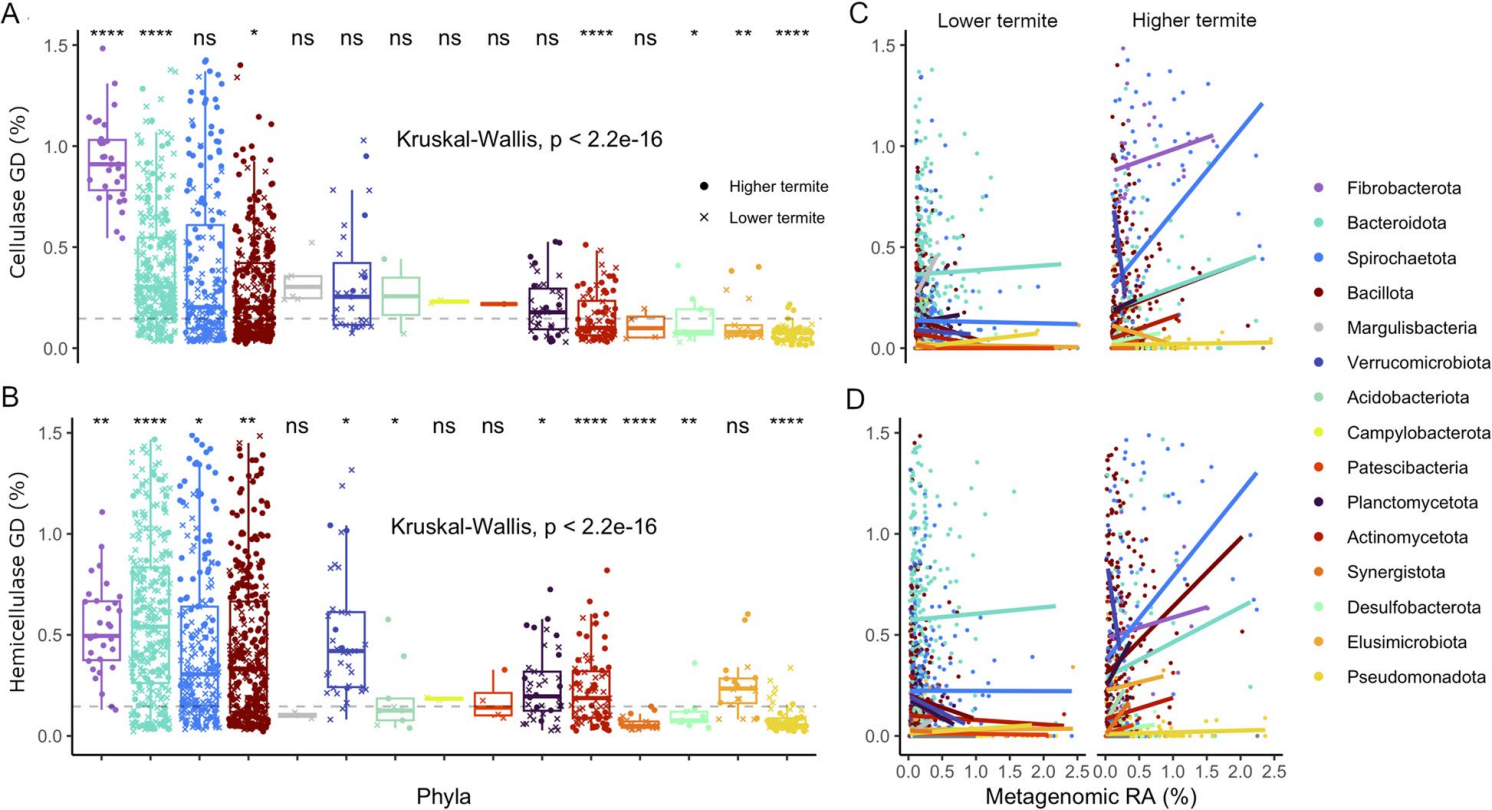
Gene density (GD) of cellulases (**A**) and hemicellulases (**B**) in the termite-associated MAGs of different bacterial phyla, and the relationships between the respective GDs and the relative abundance (RA) of the MAGs in the metagenomes of lower termites and higher termites (**C**, **D**). The number of (hemi)cellulolytic genes of a given MAG is normalized by its total number of predicted genes and expressed as percentages. Statistical tests were performed both globally and pairwise, against the mean values (dashed line). Significance values: ns = non-significant; * ≤ 0.05; ** ≤ 0.01; *** ≤ 0.001; **** ≤ 0.0001. Linear regressions are shown for all phyla but a positive correlation (Spearman ϱ > 0.1 and *p* < 0.05) was present only for LT-associated MAGs of *Bacteroidota* and HT-associated MAGs of *Fibrobacterota*, *Bacteroidota*, *Spirochaetota*, *Bacillota* and *Actinomycetota*

Using the ratio of (hemi)cellulase genes in the selected 38 CAZyme families to genes per genome (gene density, GD) to allow for abundance comparison between clades, we found that MAGs with the highest GD of cellulases were *Fibrobacterota*, followed by *Bacteroidota*, *Bacillota*, and *Spirochaetota* (Fig. 4A). The bacterial phyla with the highest hemicellulolytic potential were *Bacteroidota*, *Bacillota*, and *Spirochaetota*, followed by *Fibrobacterota* and *Verrucomicrobiota* (Fig. 4B).

**Fig. 5:**
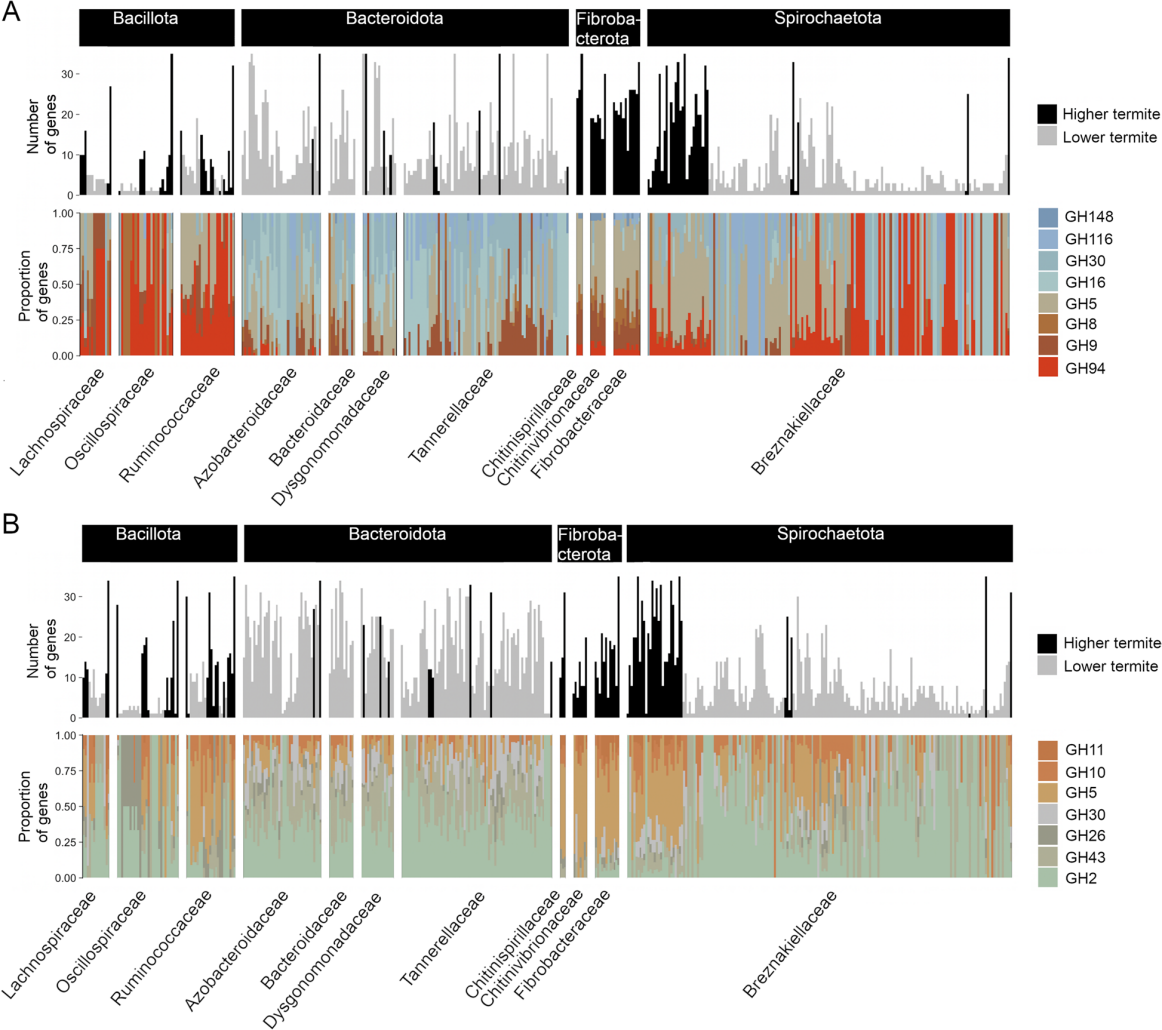
Abundance and proportion of selected CAZymes from GH families with cellulolytic (**A**) and hemicellulolytic (**B**) functions in the MAGs of selected bacterial families with a high fibrolytic potential. Each bar shows the total number of CAZymes classified as cellulase or hemicellulase that are encoded by a particular MAG (gray and black color indicates the origin from lower or higher termites) and the proportion of genes in the respective GH families (visualized with different colors). Only MAGs that encode more than one cellulase or hemicellulase were included. The families GH5 and GH30 appear twice but refer to different subfamilies classified as cellulases or hemicellulases (see Table S3 for details)

Among *Fibrobacterota*, a cluster of cellulase-containing CAZymes was highly abundant in MAGs of all families (*Fibrobacteraceae*, *Chitinivibrionaceae*, and *Chitinispirillaceae*). It is composed of GH9, GH5 (subfamilies 5, 2, and 39), GH8, and GH94 (Figs. 2 and 5A). The GH9s from our bacterial dataset comprise mainly endo-β-1,4-glucanases but only very few exo-β-1,4-glucanases, while GH8 and the specified GH5 subfamilies contain only endo-β-1,4-glucanases (Table S3). GH94 is composed mainly of enzymes with cellodextrin phosphorylase (CDP) and cellobiose phosphorylase (CBP) activity (Table S3). Although the enzymes characterized to date are generally cytoplasmic, one-third of the GH94 enzymes in our dataset have a signal peptide (Table S6). Also, MAGs of the *Oscillospiraceae*, *Lachnospiraceae*, and *Clostridiaceae* (*Bacillota*) possessed CDPs and CBPs, albeit in lower abundance (Figs. 2 and 5A).

**Fig. 6:**
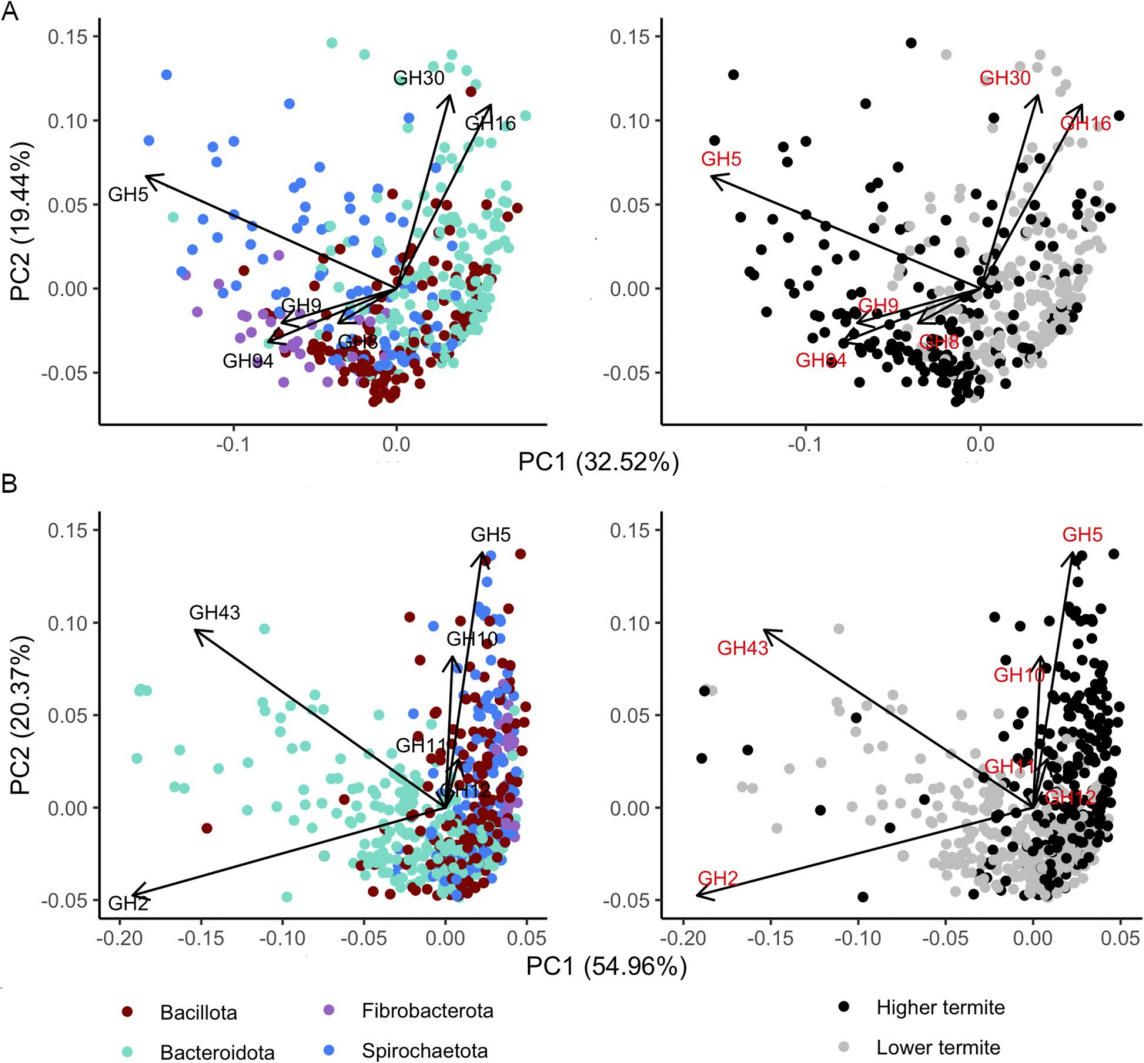
Principal component analysis (PCA) of the abundance of cellulases (**A**) and hemicellulases (**B**) in the MAGs from selected bacterial families with a high fibrolytic potential (see Fig. 5). The biplots show MAGs color-coded either according to bacterial phylum (left) or according to their respective host group (lower or higher termite; right)

The most abundant GH family in all lineages of *Bacteroidota* was GH3 (1,139 genes), corresponding to 3.3 genes per genome (GPG). Members of GH3 have β-glucosidase activity with wide substrate specificity, precluding classification as cellulases or hemicellulases (Table S3). Additionally, the orders *Bacteroidales*, *Chitinophagales*, and *Sphingobacteriales* displayed consistent abundances of GH16 (subfamilies 4, 5, and 3) and GH30 (subfamilies 1, 6, and 3; Figs. 5A and 6A). These subfamilies contained lichenases/laminarinases with endo-β-1,3- and endo-β-1,4-glucanase activity (Table S3) [98].

Among *Spirochaetota*, MAGs of *Breznakiellaceae*, *Spirochaetaceae*, and *Treponemataceae* had high abundances of GH11, GH10, GH5_22, GH5_4, and GH5_8, which contribute strongly to their divergence in hemicellulolytic functions from other phyla (Fig. 6B). Enzymes from these families have a variety of substrates, acting mainly on xyloglucans and xylomannans (Table S3). By contrast, MAGs of *Bacteroidota* showed a high abundance of GH2 (1,734 genes, 5 GPG on average). Enzymes in this family have β-galactosidase and α-arabinosidase activity (Table S3). Additionally, the MAGs from many families in the orders *Bacteroidales*, *Sphingobacteriales*, and *Chitinophagales* had GH43 genes (Figs. 2 and 5B), which encode endo-xylanase and α-arabinofuranosidase activities (Table S3). The high abundance of GH2 and GH43 genes drives the hemicellulase composition of the *Bacteroidota* MAGs (Fig. 6B), accounting for most of the observed variation. High numbers of GH2 and GH43 genes were present also among MAGs of *Verrucomicrobiota* (*Opitutaceae* and UBA953) and *Bacillota* (*Oscillospiraceae*, *Lachnospiraceae*, *Peptostreptococcaceae*, *Ruminococcaceae*) (Figs. 2 and 5B). However, *Bacillota* MAGs displayed even higher proportions of GH26, with activity on xylomannan (Fig. 5B, Table S3). While none of the CE associated with our dataset were annotated as acetylxylan esterases, we found pectin lyases and CE involved in pectin degradation (pectin acetyl esterases and pectin methyl esterases) abundantly represented in many MAGs (Tables S3, S5), particularly from the phyla *Bacteroidota* and *Planctomycetota* (Figure S6)*.*

### Differences in (hemi)cellulolytic lineages between lower and higher termites

MAGs of *Fibrobacterota* were recovered exclusively from HT, and MAGs of *Spirochaetota* MAGs from HT had a higher gene density of cellulases than those from LT (*p* < 0.001 and *r* = 0.6; Fig. 4A). There was a clear correlation between (hemi)cellulase density and metagenomic relative abundances in MAGs of *Fibrobacterota* and *Spirochaetota* from HT (both with *p* < 0.001 and *ϱ* = 0.5; Fig. 4C–D). Both *Fibrobacterota* (*Fibrobacteraceae*, *Chitinivibrionaceae*, and *Chitinispirillaceae*) and *Spirochaetota* (*Breznakiellaceae*) MAGs from HT had high proportions of GH5, GH8, and GH9 (Fig. 5A). MAGs of *Bacteroidota* and *Bacillota* differed only slightly between LT and HT (both with *p* < 0.001 and effect size *r* < 0.1; Fig. 3). However, LT-derived genomes of *Dysgonomonadaceae*, *Azobacteroidaceae*, *Bacteroidaceae*, *Tannerellaceae* (*Bacteroidota*), and *Breznakiellaceae* (*Spirochaetota*) had a higher proportion of GH30, GH16, and GH116, which act on cellodextrins (Fig. 5A).

The situation was different in the case of hemicellulases. Here, the GDs were higher in MAGs of *Spirochaetota* and *Bacillota* derived from HT (both with *p* < 0.001 and approx. *r* = 0.3; Fig. 4B), with large proportions of GH5, GH10, and GH11 (Fig. 5B). By contrast, MAGs of *Bacteroidota* from LT had higher densities of hemicellulases than those from HT (*p* < 0.001 and *r* = 0.2). LT-derived genomes of *Dysgonomonadaceae*, *Azobacteroidaceae*, *Bacteroidaceae*, *Tannerellaceae* (*Bacteroidota*), and *Breznakiellaceae* (*Spirochaetota*) had a higher proportion of GH2 and GH43, which comprise CAZymes with endoxylanase, arabinofuranosidase and β-galactosidase activities (Fig. 5B). LT-derived *Bacteroidota* MAGs with high GDs of (hemi)cellulases were abundant in the metagenomes, although the correlation between GD and relative abundance was not strong (*p* < 0.1, Spearman *ϱ* < 0.1; Fig. 4C–D). The ordination analysis reveals that the prevalence of CAZymes with different activities was driven not only by differences in taxonomy but also by their colonization patterns of LT and HT (Fig. 6).

### Carbohydrate-binding modules in CAZymes from (hemi)cellulolytic clades

Carbohydrate-binding modules (CBMs) are domains responsible for the adherence of certain CAZymes to their ligand, assuring that secreted enzymes do not diffuse away from their substrates. Among MAGs of the phylum *Fibrobacterota*, CBM4 and CBM6 were highly abundant in CAZymes from GH8, GH9, and GH5 (62 genes, average 2 GPG; 23 genes, average 0.7 GPG, respectively). Among MAGs of *Bacteroidota*, CBM6 and CBM62 were represented particularly among CAZymes from GH3 and GH2 from *Prolixibacteraceae*, *Bacteroidaceae*, *Tannerellaceae*,* Dysgonomonadaceae*, and *Azobacteroidaceae* (Fig. 2). MAGs of *Bacillota* showed a low content of CBM9, CBM22, and CBM35 (on average, max. 0.4 GPG) in CAZymes from GH2, GH43, and the GH5 subfamilies with hemicellulase activity. Cohesins, which are characteristic of the cellulosomes of *Bacillota*, were generally present only in *Oscillospiraceae* (average 1.12 GPG) but rare in *Ruminococcaceae* (average 0.3 GPG) (Table S4).

### Auxiliary activities involved in *fiber* digestion and lignin modification

**Fig. 7:**
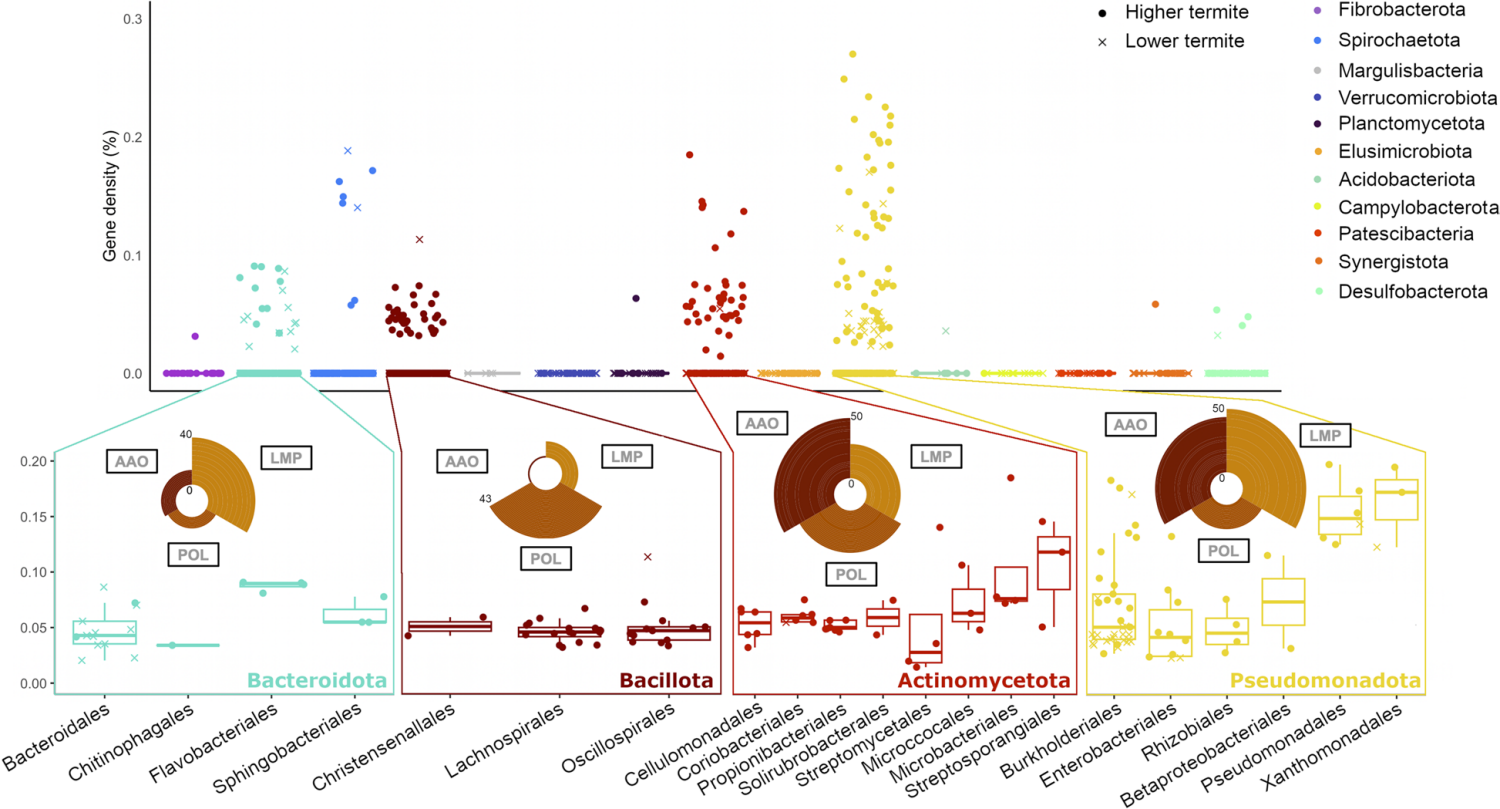
Distribution of gene densities in CAZymes with lignin-modifying activities in MAGs from different bacterial phyla. The insets break down selected phyla to the order level and show the proportion in percentages of genes from different enzyme families: POL (phenol-oxidizing laccases, AA1), LMP (lignin-modifying peroxidases, AA2), and AAO (aryl alcohol oxidases, AA3_2)

CAZyme families classified as “Auxiliary Activities” (AA) comprise different classes of oxidative enzymes that, among other substrates, can act on lignin or (hemi)cellulose (Table S3). They were less abundant than the GHs and restricted to MAGs from selected lineages (Figs. 2 and 7). Lytic cellulose/chitin monooxygenases (LPMO; AA10, 92 genes) and cellooligosaccharide dehydrogenases (CDH; AA7, 1068 genes) were most abundant among MAGs of *Pseudomonadota* (0.5 GPG on average) and *Actinobacteriota* (0.7 GPG), particularly in the orders *Burkholderiales*, *Propionibacteriales*, *Actinomycetales*, and *Microccocales* (Fig. 7).

CAZymes acting on lignin or its derivatives were also present among MAGs from *Pseudomonadota* and *Actinomycetota* (Fig. 7). They comprised phenol-oxidizing laccases (POL; AA1), lignin-modifying peroxidases (LMP; AA2), and aryl alcohol oxidases (AAO; AA3_2). CAZymes of these families were also found in MAGs from several orders of *Bacteroidota* (mainly LMP) and *Bacillota* (mainly POL) (Fig. 7). Notably, most MAGs that encode lignin-modifying activities were derived from the guts of higher termites (*p* < 0.05 and *r* = 0.25).

## Discussion

Two events in the evolutionary history of termites involved fundamental changes in the strategy of symbiotic digestion: The first event was the acquisition of cellulolytic flagellates by the common ancestor of termites and wood roaches (Cryptocercidae), which are essential for the degradation of plant fibers in lower termites (LT). The second event was the loss of these flagellates in a common ancestor of higher termites (HT), which led to functional changes that shifted lignocellulose digestion to the prokaryotic gut microbiota [5, 51].

The finding that most of the cellulolytic activity in the hindgut of *Nasutitermes* spp. (Nasutitermitinae) is associated with wood particles implicated in fiber-associated bacterial lineages in the degradation of (hemi)celluloses [6, 52]. Wood particles are colonized predominantly by members of the phyla *Fibrobacterota* (*Fibrobacterales* and *Chitinivibrioniales*) and *Spirochaetota* (mostly *Breznakiellaceae*) [14, 52]. Our results corroborate previous metagenomic and metatranscriptomic analyses of *Nasutitermes*, *Amitermes*, *Cortaritermes*, and *Microcerotermes* species [10, 14, 16, 53], extending the cellulolytic potential of *Fibrobacteraceae* and *Chitinivibrionaceae* and the hemicellulolytic potential of *Breznakiellaceae* to MAGs from almost all HT investigated, and documenting a cellulolytic potential also in MAGs of the family *Chitinispirillaceae*. The consistent presence of endo-glucanases and β-glucosidases (GH8, GH9, and certain subfamilies of GH5) — accounting for the highest cellulase gene densities of all phyla — indicates that all lineages of *Fibrobacterota* in the hindgut of HT are specialized in cellulose degradation. Conversely, high gene densities of endo-xylanases, β-xylosidases, and endo-mannanases (GH11, GH10, and certain subfamilies of GH5) identify MAGs of *Breznakiellaceae* (*Spirochaetota*) as important hemicellulose degraders in the hindgut of HT. This expands previous evidence for high expression of GH11 and GH5 transcripts in *Nasutitermes* and *Cortaritermes* sp. [10, 14], suggesting a functional specialization among the lineages of *Breznakiellaceae* associated with HT.

Notably, the (hemi)cellulolytic functions in the genomes of fiber-associated bacteria from HT resemble the activities revealed by metatranscriptomic analyses of the cellulolytic flagellates in LT [54, 55]. While the cellulolytic functions of the flagellates comprise exo-glucanases (GH7) and endo-glucanases (GH45) that were not prominent in the bacterial MAGs, the hemicellulolytic functions of the flagellates, i.e., arabinosidases (GH43), endomannases (GH26), and endo-β-1,4-xylanases (GH8, GH10 and GH11), were the same as those encoded by the fiber-associated bacteria in HT. Apparently, the loss of the (hemi)cellulolytic functions of the gut flagellates selected for bacterial lineages with different cellulolytic strategies but a similar arsenal of hemicellulolytic enzymes.

Obviously, the prediction of substrate specificity has clear limitations, and not all CAZymes in our dataset could be attributed to a specific function. This may obfuscate the results of quantitative analyses, such as phylogenetic signal or functional variability, which turned out to be remarkably similar in all instances for cellulases and hemicellulases. Furthermore, the classification of CAZymes involves simplifications, especially when enzymes that act exclusively on cellulose or hemicellulose fall into the same subfamily, or when the enzyme in question is multifunctional. Nonetheless, the fact that our qualitative analyses revealed clear differences in the subfamilies of CAZymes between particular bacterial lineages corroborates that the classifications used in our study were productive and appropriate for this dataset.

The large number of CBMs in cellulases of *Fibrobacterota* MAGs and the presence of hemicellulose-binding CBM9 in *Breznakiellaceae* MAGs are consistent with the localization of the cellulolytic and hemicellulolytic activities with the particulate fraction both in the termite gut [14, 52] and in the rumen [56]. The organization and mechanisms by which these activities are secreted are, nevertheless, still elusive in both phyla. Moreover, CBMs were encoded also by MAGs from other phyla, such as *Verrucomicrobiota*, *Bacteroidota*, and *Actinomycetota* (Fig. 2). By contrast, CBMs were rare among the diverse lineages of Clostridia with (hemi)cellulolytic potential.

The dominant cellulases in the MAGs of most clostridial lineages were cellobiose and cellodextrin phosphorylases (CBP and CDP; GH94). The CBPs and CDPs characterized to date are cytosolic enzymes that help anaerobic bacteria conserve metabolic energy by an intracellular cleavage of cellodextrins or cellobiose into glucose and glucose 1-phosphate [57, 58]. It was therefore quite unexpected that about a third of GH94 enzymes encoded by termite-associated MAGs carried a signal peptide. So far, extracellular GH94 with CBP/CDP activities have been reported only in a single proteomic study of the clostridial *Caldicellulosiruptor bescii* [59]. The presence of potentially secreted CBP/CDPs in MAGs from both LT and HT suggests that these bacteria utilize cellobiose and dextrins produced by other, cellulolytic microbiota and may explain the low number of CBMs and virtual lack of cellulosomes among the clostridial lineages.

An analysis of lignocellulose digestion by solid-state NMR has revealed significant differences in the fate of polysaccharides between wood-feeding LT and HT [60]. While both *Cryptotermes* (LT) and *Nasutitermes* (HT) efficiently degrade cellulose, the latter significantly outperforms the former in the breakdown of hemicelluloses. Apparently, the breakdown of hemicelluloses, whose structural complexity requires the concerted action of numerous enzymes [61], is accomplished more efficiently by the consortium of bacterial species that colonize the wood particles in the hindgut of HT than by the hemicellulases produced by the gut flagellates of LT [62–64]. In LT, the wood particles are sequestered into the digestive vacuoles of the flagellates as soon as they pass the enteric valve [65], precluding a primary role of gut bacteria in the breakdown of (hemi)cellulose. However, the consistent presence of β-glucosidase and cellodextrinases with CBMs in the MAGs of *Bacteroidales* associated with LT (*Bacteroidaceae*, *Dysgonomonadaceae*, *Tannerellaceae*, and *Azobacteroidaceae*) suggests that these lineages are involved in the breakdown of cellobiose and cellodextrins, which either stem from the partial degradation of amorphous cellulose by the endogenous cellulases in the midgut [66] or represent products of this incomplete digestion of cellulose by the flagellates. Such task partitioning between bacteria and flagellates had been postulated already to explain the considerable differences in the repertoire of bacterial CAZymes between LT and HT [15].

The utilization of partially digested hemicelluloses by *Bacteroidales* in LT is consistent with the compositional differences in hemicellulolytic activities to HT-associated *Breznakiellaceae* (*Spirochaetota*) and the presence of polysaccharide utilization loci (PUL) that encode both carbohydrate capture and uptake systems and CAZymes for the degradation of cellulose, xylans, and mannans in all lineages of *Bacteroidota* from termite guts [15, 67]. Notably, many of the MAGs from LT represent bacterial lineages associated with gut flagellates [22]. While the ectosymbiotic members of *Azobacteroidaceae* (*Candidatus* Symbiothrix dinenymphae),* Dysgonomonadaceae*, and *Bacteroidaceae* show the same (hemi)cellulolytic potential as their free-living relatives [68–70], the situation is different for the intracellular symbionts (*Candidatus* Azobacteroides pseudotrichonymphae) present in almost all Rhinotermidae [71, 72], whose genomes lack (hemi)cellulolytic potential [73] (Table S4).

Since the polyphenolic component of lignocellulose provides a major obstacle to the enzymatic attack of cellulose and hemicelluloses, the fate of lignin during termite gut passage has been disputed for decades [74]. The mechanistic challenges of lignin degradation are rooted in the stability of the inter-monomeric linkages, which cannot be hydrolyzed but require oxidative attack by (per)oxidases [75]. Meanwhile, there is compelling evidence that lignin is modified during gut passage in all termites investigated [76–79]. Nonetheless, there are fundamental differences between LT and HT. While LT disrupts the covalent bonds between hemicelluloses and lignin, leaving the latter largely intact, HT efficiently degrade the lignin polymer, as evidenced by the depletion of major inter-unit linkages and methoxyls [78, 79].

The capacity for the breakdown of lignin is found in many soil bacteria [81], which possess the same types of extracellular (per)oxidases that are present in lignin-degrading fungi [75, 81, 82]. Many of these lineages were represented among our termite MAGs, particularly within *Actinomycetota* (*Micrococcales*, *Actinomycetales*, and *Mycobacteriales*) and *Pseudomonadota* (*Burkholderiales* and *Pseudomonadales*). The MAGs of each lineage encoded a diverse set of laccases, lignin peroxidases, and aryl-alcohol oxidases. Laccases (phenol oxidases) are the most important agents of bacterial lignin degradation that not only oxidize phenolic substrates but — in the presence of lignin degradation products as mediator — also cleave the inter-unit bonds of the lignin polymer [83]. The same bonds are cleaved also by lignin peroxidases, which require hydrogen peroxide produced by aryl-alcohol oxidases as co-substrate [75].

It is noteworthy that almost all MAGs with lignin-modifying capacity were recovered from HT (Fig. 7). In LT, the situation is reminiscent of lignocellulose degradation by brown rot fungi, which employ Fenton chemistry to selectively metabolize carbohydrates without significant lignin removal, where the extracellular production of Fe^2+^ and H_2_O_2_ leads to the formation of hydroxyl radicals [84]. It has been proposed that laccases produced in the salivary glands of the host also contribute to delignification during midgut passage in LT [85].

Several lineages of MAGs assigned to *Actinobacteriota* and *Pseudomonadota* encode lytic cellulose monooxygenase (LPMO) and cellobiose dehydrogenase (CDH). LPMOs are highly effective in the depolymerization of recalcitrant polysaccharides because they cleave crystalline cellulose, generating free ends that are accessible to exoglucanases [87, 88]. CDHs shuttle electrons from the oxidation of cellobiose to the catalytic site of LPMO [89]. A metatranscriptomic analysis of a *Labiotermes* sp. has documented that bacterial LPMOs are expressed mainly in the anterior hindgut [11]. It is possible that the oxygen-dependent activities of *Actinomycetales* and *Pseudomonadales* in the hindgut of HT enhance the degradation of wood fibers by polysaccharide-digesting bacteria. However, the small proportion of exoglucanases in the hindgut microbiota of higher termites may have a different explanation. Many endoglucanases, particularly in GH5 and GH9, are processive and have been shown to attack both microcrystalline and amorphous regions of cellulose, producing cellobiose, cellotriose, and cellotetraose as final products [80]. Hence, it is plausible that fiber-associated bacteria can effectively hydrolyze cellulose fibers with the synergistic action of their diverse endoglucanases, as already demonstrated for *Fibrobacter succinogenes*, the most important fiber-degrading bacterium in the rumen [86]. A recent report on a GH5_4 cellulase with high activities on crystalline cellulose from the metagenome of the camel rumen [99] illustrates that further insights into the mechanism of crystalline cellulase degradation by the hindgut bacteria of termites will be gained by integrating both bioinformatics and enzymology.

Both oxidases and oxygenases, and indirectly also peroxidases, require molecular oxygen as co-substrate. Microsensor measurements have revealed a strong influx of oxygen across the termite gut epithelium, creating microoxic habitats in the periphery of an otherwise anoxic hindgut [90]. Notably, MAGs encoding lignin-modifying enzymes belonged to the same bacterial lineages that were localized at the internal surface of the hindgut wall. Fluorescence in situ hybridization (FISH) has revealed that the microoxic periphery of the hindgut of *Mastotermes darwiniensis* (LT) is colonized by *Alphaproteobacteria* and *Betaproteobacteria* (*Pseudomonadota*) [91]*.* Clone libraries of the hindgut contents of *Reticulitermes santonensis* and *Reticulitermes speratus* (LT) and a *Nasutitermes* spp. (HT) have identified *Actinomycetota*, *Bacteroidota*, *Pseudomonadota,* and *Spirochaetota* phylotypes in the wall fraction [92–94]. They include the same lineages of *Actinomycetales*, *Corynebacteriales*, and *Propionibacteriales* that are represented by our MAGs with lignin- and cellulose-oxidizing potential (Fig. 7).

## Conclusions

Previous studies revealed unique patterns of bacterial community structure of termites that were attributed to differences in host phylogeny, diet, and microenvironmental factors [95–97]. Our results altogether indicate that this distribution of specific bacterial lineages determines the CAZyme patterns among different host groups. Many members of the bacterial gut microbiota, such as major lineages in the phyla *Elusimicrobiota* and *Patescibacteria*, have a very low content of GHs, indicating they do not contribute to the degradation of polysaccharides and possess only CAZymes necessary for assimilatory functions, such as cell wall maintenance. Many lineages with pronounced (hemi)cellulytic capacities, such as certain *Bacteroidales* families, occur only in LT, whereas others, such as *Fibrobacterota* and *Actinomycetota*, were present only in HT. In *Spirochaetota*, the fibrolytic capacities differ strongly between lineages from LT and HT, and *Actinomycetota* and *Pseudomonadota* with fibrolytic functions occur only in HT.

Although the reasons for these patterns and their implications for the nutritional symbioses in termite guts seem to be multifactorial and complex, they are ultimately driven by the presence of flagellates in LT and their subsequent loss in HT. In LT, where lignin modification seems to be restricted to host activities in the midgut, the flagellates efficiently depolymerize cellulose but accomplish only incomplete digestion of hemicelluloses, and free-living or flagellate-associated *Bacteroidales* and *Breznakiellaceae* exploit residual cellodextrins and hemicelluloses. In HT, however, the degradation of lignocellulose is partitioned among a consortium of bacterial lineages with different capabilities. *Pseudomonadota* and *Actinomycetota* that colonize the microoxic periphery of the hindgut partially depolymerize lignin and generate free-ends in the crystalline regions of cellulose using oxygen-dependent activities. This increases the accessibility of the plant fibers for *Fibrobacterota* and HT-specific *Breznakiellaceae*, which possess sophisticated fiber-adhesion mechanisms and effectively depolymerize high-molecular-weight cellulose and hemicelluloses. Finally, the low-molecular remains of (hemi)celluloses released by the activities of the fiber-associated microbiota are processed by various bacterial lineages, including several families of *Clostridia*, which seem to employ hitherto unrecognized strategies involving extracellular cellobiose and cellodextrin phosphorylases.

## Supplementary Information

Additional file 1: Table S1. Termite gut metagenomes metadata. Table S2. MAG classification, metagenomic relative abundance, and quality statistics. Table S3. Functional classification and activity of CAZyme (sub)families represented in the MAGs analyzed in this study. Table S4. Genomic abundance of CAZymes with lignocellulolytic activity in each MAG of the bacterial lineages depicted in Figure 2. Table S5. Genomic abundance of all CAZymes across all MAGs included in this study. Table S6. Prediction of secretion fate for all GH94 genes and the taxonomy of the respective MAGs.

Additional file 2: Figure S1. UMAP biplot of the distribution of CAZymes of termite-associated bacteria color-coded according to phylum in (A) lower termites, (B) higher termites, (C) termite diet, and (D) gut compartment. Figure S2. Distribution of phylogenetic signal across termite-associated bacterial phyla tested against random effects. Figure S3. The drivers of uniqueness in the distribution patterns of (hemi)cellulases and lignin-modification enzymes in 2,223 termite-associated bacterial MAGs. Figure S4. The gene density of cellulases (A) and hemicellulases (B) among the MAGs of *Firmicutes*, *Spirochaetota*,*Fibrobacterota*, and *Bacteroidota* from lower and higher termites. Figure S5. Biplot of the PCA based on the distribution of lignin-modifying enzymes, color coded according to the phylum to which each genome belongs (A) and according to the gut compartment sampled for the metagenomes (B). Figure S6. Distribution of gene densities in CAZymes with pectinase activities in MAGs from different bacterial phyla. The insets break down the two most abundant phyla, *Bacteroidota* and *Planctomycetota*, to the order level.

## Data Availability

The MAGs used in this study are available in the NCBI Sequence Read Archive (SRA) under the BioProject accession numbers PRJNA560329 and PRJNA732531. All data generated or analyzed during this study are included in this published article and its supplementary information files.

## References

[1] Chouvenc T, Šobotník J, Engel MS, Bourguignon T. Termite evolution: mutualistic associations, key innovations, and the rise of Termitidae. Cell Mol Life Sci. 2021;78:2749–69. 33388854 10.1007/s00018-020-03728-z PMC11071720

[2] Ni J, Tokuda G. Lignocellulose-degrading enzymes from termites and their symbiotic microbiota. Biotechnol Adv. 2013;31:838–50. 23623853 10.1016/j.biotechadv.2013.04.005

[3] Tokuda, G. Plant cell wall degradation in insects: recent progress on endogenous enzymes revealed by multi-omics technologies. In: Jurenka, R. editor. *Advances in Insect Physiology*. Cambridge: Academic Press; 2019. vol. 57. p. 97–136

[4] Brune A. Symbiotic digestion of lignocellulose in termite guts. Nat Rev Microbiol. 2014;12:168–80. 24487819 10.1038/nrmicro3182

[5] Brune A, Dietrich C. The gut microbiota of termites: digesting the diversity in the light of ecology and evolution. Annu Rev Microbiol. 2015;69:145–66. 26195303 10.1146/annurev-micro-092412-155715

[6] Tokuda G, Watanabe H. Hidden cellulases in termites: revision of an old hypothesis. Biol Lett. 2007;3:336–9. 17374589 10.1098/rsbl.2007.0073 PMC2464699

[7] Warnecke F, Luginbühl P, Ivanova N, Ghassemian M, Richardson TH, Stege JT, et al. Metagenomic and functional analysis of hindgut microbiota of a wood-feeding higher termite. Nature. 2007;450:560–5. 18033299 10.1038/nature06269

[8] Schalk, F., Gostinčar, C., Kreuzenbeck, N. B., Conlon, B. H., Sommerwerk, E., Rabe, P., *et al.* The termite fungal cultivar *Termitomyces* combines diverse enzymes and oxidative reactions for plant biomass conversion. *mBio*. 2021;12:e0355120. 10.1128/mBio.03551-20 PMC8262964 34126770

[9] Li H, Young SE, Poulsen M, Currie CR. Symbiont-mediated digestion of plant biomass in fungus-farming insects. Annu Rev Entomol. 2021;66:297–316. 32926791 10.1146/annurev-ento-040920-061140

[10] Calusinska M, Marynowska M, Bertucci M, Untereiner B, Klimek D, Goux X, et al. Integrative omics analysis of the termite gut system adaptation to *Miscanthus* diet identifies lignocellulose degradation enzymes. Commun Biol. 2020;3:1–12. 32483294 10.1038/s42003-020-1004-3 PMC7264248

[11] Marynowska M, Sillam-Dussès D, Untereiner B, Klimek D, Goux X, Gawron P, et al. A holobiont approach towards polysaccharide degradation by the highly compartmentalised gut system of the soil-feeding higher termite *Labiotermes labralis*. BMC Genomics. 2023;24:115. 36922761 10.1186/s12864-023-09224-5 PMC10018900

[12] Romero Victorica M, Soria MA, Batista-García RA, Ceja-Navarro JA, Vikram S, Ortiz M, et al. Neotropical termite microbiomes as sources of novel plant cell wall degrading enzymes. Sci Rep. 2020;10:3864. 32123275 10.1038/s41598-020-60850-5 PMC7052144

[13] Guerrero EB, de Villegas RMD, Soria MA, Santangelo MP, Campos E, Talia PM. Characterization of two GH5 endoglucanases from termite microbiome using synthetic metagenomics. Appl Microbiol Biotechnol. 2020;104:8351–66. 32816085 10.1007/s00253-020-10831-5

[14] Tokuda G, Mikaelyan A, Fukui C, Matsuura Y, Watanabe H, Fujishima M, et al. Fiber-associated spirochetes are major agents of hemicellulose degradation in the hindgut of wood-feeding higher termites. Proc Natl Acad Sci. 2018;115:E11996–2004. 30504145 10.1073/pnas.1810550115 PMC6304966

[15] Arora J, Kinjo Y, Šobotník J, Buček A, Clitheroe C, Stiblik P, et al. The functional evolution of termite gut microbiota. Microbiome. 2022;10:78. 35624491 10.1186/s40168-022-01258-3 PMC9137090

[16] Rahman, N., Parks, D. H., Vanwonterghem, I., Morrison, M., Tyson, G. W. & Hugenholtz, P. A phylogenomic analysis of the bacterial phylum *Fibrobacteres*. *Front. Microbiol.* 2016;6; 10.3389/fmicb.2015.01469. 10.3389/fmicb.2015.01469 PMC4704652 26779135

[17] Levasseur A, Drula E, Lombard V, Coutinho PM, Henrissat B. Expansion of the enzymatic repertoire of the CAZy database to integrate auxiliary redox enzymes. Biotechnol Biofuels. 2013;6:41. 23514094 10.1186/1754-6834-6-41 PMC3620520

[18] Armendáriz-Ruiz M, Rodríguez-González JA, Camacho-Ruíz RM, Mateos-Díaz JC. Carbohydrate esterases: an overview. Methods Mol Biol Clifton NJ. 2018;1835:39–68. 10.1007/978-1-4939-8672-9_2 30109645

[19] Garron M-L, Cygler M. Structural and mechanistic classification of uronic acid-containing polysaccharide lyases. Glycobiology. 2010;20:1547–73. 20805221 10.1093/glycob/cwq122

[20] Lairson LL, Henrissat B, Davies GJ, Withers SG. Glycosyltransferases: structures, functions, and mechanisms. Annu Rev Biochem. 2008;77:521–55. 18518825 10.1146/annurev.biochem.76.061005.092322

[21] Hervé V, Liu P, Dietrich C, Sillam-Dussès D, Stiblik P, Šobotník J, et al. Phylogenomic analysis of 589 metagenome-assembled genomes encompassing all major prokaryotic lineages from the gut of higher termites. PeerJ. 2020;8: e8614. 32095380 10.7717/peerj.8614 PMC7024585

[22] Mies, U. S., Hervé, V., Kropp, T., Platt, K., Sillam-Dussès, D., Šobotník, J., *et al.* Genome reduction and horizontal gene transfer in the evolution of *Endomicrobia*—rise and fall of an intracellular symbiosis with termite gut flagellates. mBio*.* 2024;0: e00826–24. 10.1128/mbio.00826-24 PMC11257099 38742878

[23] Parks DH, Imelfort M, Skennerton CT, Hugenholtz P, Tyson GW. CheckM: assessing the quality of microbial genomes recovered from isolates, single cells, and metagenomes. Genome Res. 2015;25:1043–55. 25977477 10.1101/gr.186072.114 PMC4484387

[24] Chaumeil P-A, Mussig AJ, Hugenholtz P, Parks DH. GTDB-Tk v2: memory friendly classification with the genome taxonomy database. Bioinformatics. 2022;38:5315–6. 36218463 10.1093/bioinformatics/btac672 PMC9710552

[25] Nguyen L-T, Schmidt HA, von Haeseler A, Minh BQ. IQ-TREE: a fast and effective stochastic algorithm for estimating maximum-likelihood phylogenies. Mol Biol Evol. 2015;32:268–74. 25371430 10.1093/molbev/msu300 PMC4271533

[26] Matsen FA, Kodner RB, Armbrust EV. Pplacer: linear time maximum-likelihood and Bayesian phylogenetic placement of sequences onto a fixed reference tree. BMC Bioinformatics. 2010;11:538. 21034504 10.1186/1471-2105-11-538 PMC3098090

[27] Romero Arias J, Boom A, Wang M, Clitheroe C, Šobotník J, Stiblik P, et al. Molecular phylogeny and historical biogeography of Apicotermitinae (Blattodea: Termitidae). Syst Entomol. 2021;46:741–56.

[28] Wang M, Hellemans S, Šobotník J, Arora J, Buček A, Sillam-Dussès D, et al. Phylogeny, biogeography and classification of Teletisoptera (Blattaria: Isoptera). Syst Entomol. 2022;47:581–90.

[29] Paradis E, Claude J, Strimmer K. APE: analyses of phylogenetics and evolution in R language. Bioinformatics. 2004;20:289–90. 14734327 10.1093/bioinformatics/btg412

[30] Yang Z. PAML: a program package for phylogenetic analysis by maximum likelihood. Bioinformatics. 1997;13:555–6. 10.1093/bioinformatics/13.5.555 9367129

[31] Letunic I, Bork P. Interactive Tree Of Life (iTOL) v5: an online tool for phylogenetic tree display and annotation. Nucleic Acids Res. 2021;49:W293–6. 33885785 10.1093/nar/gkab301 PMC8265157

[32] Xu S, Li L, Luo X, Chen M, Tang W, Zhan L, et al. Ggtree: a serialized data object for visualization of a phylogenetic tree and annotation data. iMeta. 2022;1(e56). 10.1002/imt2.56 PMC10989815 38867905

[33] Hyatt D, Chen G-L, LoCascio PF, Land ML, Larimer FW, Hauser LJ. Prodigal: prokaryotic gene recognition and translation initiation site identification. BMC Bioinformatics. 2010;11:119. 20211023 10.1186/1471-2105-11-119 PMC2848648

[34] Haft DH, Selengut JD, White O. The TIGRFAMs database of protein families. Nucleic Acids Res. 2003;31:371–3. 12520025 10.1093/nar/gkg128 PMC165575

[35] Mistry J, Chuguransky S, Williams L, Qureshi M, Salazar GA, Sonnhammer ELL, et al. Pfam: the protein families database in 2021. Nucleic Acids Res. 2021;49:D412–9. 33125078 10.1093/nar/gkaa913 PMC7779014

[36] Pandurangan AP, Stahlhacke J, Oates ME, Smithers B, Gough J. The SUPERFAMILY 2.0 database: a significant proteome update and a new webserver. Nucleic Acids Res. 2019;47:D490–4. 30445555 10.1093/nar/gky1130 PMC6324026

[37] Mi H, Muruganujan A, Casagrande JT, Thomas PD. Large-scale gene function analysis with the PANTHER classification system. Nat Protoc. 2013;8:1551–66. 23868073 10.1038/nprot.2013.092 PMC6519453

[38] Marchler-Bauer A, Derbyshire MK, Gonzales NR, Lu S, Chitsaz F, Geer LY, et al. CDD: NCBI’s conserved domain database. Nucleic Acids Res. 2015;43:D222–6. 25414356 10.1093/nar/gku1221 PMC4383992

[39] Jones P, Binns D, Chang H-Y, Fraser M, Li W, McAnulla C, et al. InterProScan 5: genome-scale protein function classification. Bioinformatics. 2014;30:1236–40. 24451626 10.1093/bioinformatics/btu031 PMC3998142

[40] Zheng J, Ge Q, Yan Y, Zhang X, Huang L, Yin Y. dbCAN3: automated carbohydrate-active enzyme and substrate annotation. Nucleic Acids Res. 2023;51:W115–21. 37125649 10.1093/nar/gkad328 PMC10320055

[41] Buchfink B, Xie C, Huson DH. Fast and sensitive protein alignment using DIAMOND. Nat Methods. 2015;12:59–60. 25402007 10.1038/nmeth.3176

[42] Drula E, Garron M-L, Dogan S, Lombard V, Henrissat B, Terrapon N. The carbohydrate-active enzyme database: functions and literature. Nucleic Acids Res. 2022;50:D571–7. 34850161 10.1093/nar/gkab1045 PMC8728194

[43] Teufel F, Almagro Armentero JJ, Johansen AR, Gíslason MH, Pihl SI, Tsirigos KD. SignalP 6.0 predicts all five types of signal peptides using protein language models. Nat Biotechnol. 2022;40:1023–5. 34980915 10.1038/s41587-021-01156-3 PMC9287161

[44] Käll L, Krogh A, Sonnhammer ELL. Advantages of combined transmembrane topology and signal peptide prediction—the Phobius web server. Nucleic Acids Res. 2007;35:W429–32. 17483518 10.1093/nar/gkm256 PMC1933244

[45] McInnes L, Healy J, Saul N, Großberger L. UMAP: uniform manifold approximation and projection. J Open Source Softw. 2018;3:861.

[46] Oksanen, J., Blanchet, G., Friendly, M., Kindt, R., Legendre, P., McGlinn, D., *et al.* Vegan: community ecology package. 2019; https://cran.r-project.org/package=vegan

[47] R Core Team. R: A language and environment for statistical computing. 2013.

[48] Fox, J. & Weisberg, S. An R companion to applied regression. *SAGE Publications Inc.* 2024;https://us.sagepub.com/en-us/nam/an-r-companion-to-applied-regression/book246125.

[49] Kassambara, A. rstatix: pipe-friendly framework for basic statistical tests. 2023; https://rpkgs.datanovia.com/rstatix/.

[50] Keck F, Rimet F, Bouchez A, Franc A. Phylosignal: an R package to measure, test, and explore the phylogenetic signal. Ecol Evol. 2016;6:2774–80. 27066252 10.1002/ece3.2051 PMC4799788

[51] Ohkuma, M. & Brune, A. Diversity, structure, and evolution of the termite gut microbial community. In: Bignell, D. E., Roisin, Y. & Lo, N., editors. *Biology of Termites: a Modern Synthesis.* Dordrecht: Springer; 2011; 10.1007/978-90-481-3977-4_15.

[52] Mikaelyan A, Strassert JFH, Tokuda G, Brune A. The fibre-associated cellulolytic bacterial community in the hindgut of wood-feeding higher termites (Nasutitermes spp.). Environ Microbiol. 2014;16:2711–22.

[53] He S, Ivanova N, Kirton E, Allgaier M, Bergin C, Scheffrahn RH. Comparative metagenomic and metatranscriptomic analysis of hindgut paunch microbiota in wood- and dung-feeding higher termites. Plos one. 2013;8:e61126. 23593407 10.1371/journal.pone.0061126 PMC3625147

[54] Todaka N, Inoue T, Saita K, Ohkuma M, Nalepa CA, Lenz M, et al. Phylogenetic analysis of cellulolytic enzyme genes from representative lineages of termites and a related cockroach. PLoS ONE. 2010;5: e8636. 20072608 10.1371/journal.pone.0008636 PMC2797642

[55] Todaka N, Moriya S, Saita K, Hondo T, Kiuchi I, Takasu H, et al. Environmental cDNA analysis of the genes involved in lignocellulose digestion in the symbiotic protist community of *Reticulitermes speratus*. FEMS Microbiol Ecol. 2007;59:592–9. 17239084 10.1111/j.1574-6941.2006.00237.x

[56] Hess M, Sczyrba A, Egan R, Kim T-W, Chokhawala H, Schroth G, et al. Metagenomic discovery of biomass-degrading genes and genomes from cow rumen. Science. 2011;331:463–7. 21273488 10.1126/science.1200387

[57] Liu N, Fosses A, Kampik C, Parsiegla G, Denis Y, Vita N, et al. In vitro and in vivo exploration of the cellobiose and cellodextrin phosphorylases panel in *Ruminiclostridium cellulolyticum*: implication for cellulose catabolism. Biotechnol Biofuels. 2019;12:208. 31497068 10.1186/s13068-019-1549-x PMC6720390

[58] Lou J, Dawson KA, Strobel HJ. Role of phosphorolytic cleavage in cellobiose and cellodextrin metabolism by the ruminal bacterium *Prevotella ruminicola*. Appl Environ Microbiol. 1996;62:1770–3. 8633876 10.1128/aem.62.5.1770-1773.1996 PMC167952

[59] Poudel S, Giannone RJ, Basen M, Nookaew I, Poole FL, Kelly RM, et al. The diversity and specificity of the extracellular proteome in the cellulolytic bacterium *Caldicellulosiruptor bescii* is driven by the nature of the cellulosic growth substrate. Biotechnol Biofuels. 2018;11:80. 29588665 10.1186/s13068-018-1076-1 PMC5865380

[60] Xue Y, Li H, Kang X. Molecular unraveling of polysaccharide digestion in wood-feeding termites: a solid-state NMR perspective. Carbohydr Polym. 2024;331: 121843. 38388031 10.1016/j.carbpol.2024.121843

[61] Houfani AA, Anders N, Spiess AC, Baldrian P, Benallaoua S. Insights from enzymatic degradation of cellulose and hemicellulose to fermentable sugars–a review. Biomass Bioenergy. 2020;134: 105481.

[62] Franco Cairo JPL, Mandelli F, Tramontina R, Cannella D, Paradisi A, Ciano L., et al. Oxidative cleavage of polysaccharides by a termite-derived superoxide dismutase boosts the degradation of biomass by glycoside hydrolases. *Green Chem.* Int J Green Chem Res GC 2022;24,4845–58. 10.1039/d1gc04519a PMC9208272 35813357

[63] Xie L, Zhang L, Zhong Y, Liu N, Long Y, Wang S, et al. Profiling the metatranscriptome of the protistan community in *Coptotermes formosanus* with emphasis on the lignocellulolytic system. Genomics. 2012;99:246–55. 22326742 10.1016/j.ygeno.2012.01.009

[64] Nishimura Y, Otagiri M, Yuki M, Shimizu M, Inoue J, Moriya S, et al. Division of functional roles for termite gut protists revealed by single-cell transcriptomes. ISME J. 2020;14:2449–60. 32514117 10.1038/s41396-020-0698-z PMC7490689

[65] Yoshimura T, Fujino T, Itoh T, Tsunoda K, Takahashi M. Ingestion and decomposition of wood and cellulose by the protozoa in the hindgut of *Coptotermes formosanus* Shiraki (Isoptera: Rhinotermitidae) as evidenced by polarizing and transmission electron microscopy. Holzforschung. 1996;50:99–104.

[66] Lo, N., Tokuda, G. & Watanabe, H. Evolution and function of endogenous termite cellulases. In: Bignell, D. E., Roisin, Y. & Lo, N., editors. *Biology of Termites: a Modern Synthesis*. Dordrecht: Springer; 2011; 10.1007/978-90-481-3977-4_3.

[67] Arnal G, Bastien G, Monties N, Abot A, Anton Leberre V, Bozonnet S, et al. Investigating the function of an arabinan utilization locus isolated from a termite gut Community. Appl Environ Microbiol. 2015;81:31–9. 25304507 10.1128/AEM.02257-14 PMC4272722

[68] Noda S, Hongoh Y, Sato T, Ohkuma M. Complex coevolutionary history of symbiotic *Bacteroidales* bacteria of various protists in the gut of termites. BMC Evol Biol. 2009;9:158. 19586555 10.1186/1471-2148-9-158 PMC2717939

[69] Treitli SC, Kolisko M, Husník F, Keeling PJ, Hampl V. Revealing the metabolic capacity of *Streblomastix strix* and its bacterial symbionts using single-cell metagenomics. Proc Natl Acad Sci. 2019;116:19675–84. 31492817 10.1073/pnas.1910793116 PMC6765251

[70] Yuki M, Kuwahara H, Shintani M, Izawa K, Sato T, Starns D, et al. Dominant ectosymbiotic bacteria of cellulolytic protists in the termite gut also have the potential to digest lignocellulose. Environ Microbiol. 2015;17:4942–53. 26079531 10.1111/1462-2920.12945

[71] Noda S, Iida T, Kitade O, Nakajima H, Kudo T, Ohkuma M. Endosymbiotic *Bacteroidales* bacteria of the flagellated protist *Pseudotrichonympha grassii* in the gut of the termite *Coptotermes formosanus*. Appl Environ Microbiol. 2005;71:8811–7. 16332877 10.1128/AEM.71.12.8811-8817.2005 PMC1317455

[72] Noda S, Kitade O, Inoue T, Kawai M, Kanuka M, Hiroshima K, et al. Cospeciation in the triplex symbiosis of termite gut protiststheir hosts, and their bacterial endosymbionts. Mol Ecol. 2007;16:1257–66. 17391411 10.1111/j.1365-294X.2006.03219.x

[73] Hongoh Y, Sharma VK, Prakash T, Noda S, Toh H, Taylor TD, et al. Genome of an endosymbiont coupling N2 fixation to cellulolysis within protist cells in termite gut. Science. 2008;322:1108–9. 19008447 10.1126/science.1165578

[74] Breznak JA, Brune A. Role of microorganisms in the digestion of lignocellulose by termites. Annu Rev Entomol. 1994;39:453–87.

[75] Janusz G, Pawlik A, Sulej J, Świderska-Burek U, Jarosz-Wilkołazka A, Paszczyński A. Lignin degradation: microorganisms, enzymes involved, genomes analysis and evolution. FEMS Microbiol Rev. 2017;41:941–62. 29088355 10.1093/femsre/fux049 PMC5812493

[76] Geib, S. M., Filley, T. R., Hatcher, P. G., Hoover, K., Carlson, J. E., Jimenez-Gasco, M. del M., *et al.* Lignin degradation in wood-feeding insects. *Proc. Natl. Acad. Sci.* 2008;105:12932–7. 10.1073/pnas.0805257105 PMC2529026 18725643

[77] Li H, Yelle DJ, Li C, Yang M, Ke J, Zhang R, et al. Lignocellulose pretreatment in a fungus-cultivating termite. Proc Natl Acad Sci. 2017;114:4709–14. 28424249 10.1073/pnas.1618360114 PMC5422824

[78] Li, H., Kang, X., Yang, M., Kasseney, B. D., Zhou, X., Liang, S., *et al.* Molecular insights into the evolution of woody plant decay in the gut of termites. *Sci. Adv.* 2023;9: eadg1258. 10.1126/sciadv.adg1258 PMC10208576 37224258

[79] Tarmadi D, Tobimatsu Y, Yamamura M, Miyamoto T, Miyagawa Y, Umezawa T, et al. NMR studies on lignocellulose deconstructions in the digestive system of the lower termite *Coptotermes formosanus* Shiraki. Sci Rep. 2018;8:1290. 29358744 10.1038/s41598-018-19562-0 PMC5778066

[80] Wu S, Wu S. Processivity and the mechanisms of processive endoglucanases. Appl Biochem Biotechnol. 2020;190:448–63. 31378843 10.1007/s12010-019-03096-w

[81] Tian J-H, Pourcher A-M, Bouchez T, Gelhaye E, Peu P. Occurrence of lignin degradation genotypes and phenotypes among prokaryotes. Appl Microbiol Biotechnol. 2014;98:9527–44. 25343973 10.1007/s00253-014-6142-4

[82] Silva JP, Ticona ARP, Hamann PRV, Quirino BF, Noronha EF. Deconstruction of lignin: from enzymes to microorganisms. Molecules. 2021;26:2299. 33921125 10.3390/molecules26082299 PMC8071518

[83] Munk L, Sitarz AK, Kalyani DC, Mikkelsen JD, Meyer AS. Can laccases catalyze bond cleavage in lignin? Biotechnol Adv. 2015;33:13–24. 25560931 10.1016/j.biotechadv.2014.12.008

[84] Martinez D, Challacombe J, Morgenstern I, Hibbett D, Schmoll M, Kubicek CP, et al. Genome, transcriptome, and secretome analysis of wood decay fungus *Postia placenta* supports unique mechanisms of lignocellulose conversion. Proc Natl Acad Sci. 2009;106:1954–9. 19193860 10.1073/pnas.0809575106 PMC2644145

[85] Coy MR, Salem TZ, Denton JS, Kovaleva ES, Liu Z, Barber DS, et al. Phenol-oxidizing laccases from the termite gut. Insect Biochem Mol Biol. 2010;40:723–32. 20691784 10.1016/j.ibmb.2010.07.004

[86] Qi, M., Jun, H., Forsberg, C.W. Characterization and synergistic interactions of *Fibrobacter succinogenes* glycoside hydrolases. *Appl Environ Microbiol*. 2007;73. 10.1128/AEM.01037-07 10.1128/AEM.01037-07 PMC2075001 17660301

[87] Franco Cairo JPL, Cannella D, Oliveira LC, Gonçalves TA, Rubio MV, Terrasan CRF, et al. On the roles of AA15 lytic polysaccharide monooxygenases derived from the termite *Coptotermes gestroi*. J Inorg Biochem. 2021;216: 111316. 33421883 10.1016/j.jinorgbio.2020.111316

[88] Vaaje-Kolstad G, Westereng B, Horn SJ, Liu Z, Zhai H, Sørlie M, et al. An oxidative enzyme boosting the enzymatic conversion of recalcitrant polysaccharides. Science. 2010;330:219–22. 20929773 10.1126/science.1192231

[89] Tan T-C, Kracher D, Gandini R, Sygmund C, Kittl R, Haltrich D, et al. Structural basis for cellobiose dehydrogenase action during oxidative cellulose degradation. Nat Commun. 2015;6:7542. 26151670 10.1038/ncomms8542 PMC4507011

[90] Brune A, Emerson D, Breznak JA. The termite gut microflora as an oxygen sink: microelectrode determination of oxygen and pH gradients in guts of lower and higher termites. Appl Environ Microbiol. 1995;61:2681–7. 16535076 10.1128/aem.61.7.2681-2687.1995 PMC1388494

[91] Berchtold M, Chatzinotas A, Schönhuber W, Brune A, Amann R, Hahn D, et al. Differential enumeration and in situ localization of microorganisms in the hindgut of the lower termite *Mastotermes darwiniensis* by hybridization with rRNA-targeted probes. Arch Microbiol. 1999;172:407–16. 10591851 10.1007/s002030050778

[92] Nakajima H, Hongoh Y, Usami R, Kudo T, Ohkuma M. Spatial distribution of bacterial phylotypes in the gut of the termite *Reticulitermes speratus* and the bacterial community colonizing the gut epithelium. FEMS Microbiol Ecol. 2005;54:247–55. 16332323 10.1016/j.femsec.2005.03.010

[93] Yang H, Schmitt-Wagner D, Stingl U, Brune A. Niche heterogeneity determines bacterial community structure in the termite gut (*Reticulitermes santonensis*). Environ Microbiol. 2005;7:916–32. 15946289 10.1111/j.1462-2920.2005.00760.x

[94] Köhler, T., Dietrich, C., Scheffrahn, R. H. & Brune, A. high-resolution analysis of gut environment and bacterial microbiota reveals functional compartmentation of the gut in wood-feeding higher termites (*Nasutitermes* spp.). *Appl. Environ. Microbiol.* 2012;78:4691–701. 10.1128/AEM.00683-12 PMC3370480 22544239

[95] Rossmassler K, Dietrich C, Thompson C, Mikaelyan A, Nonoh JO, Scheffrahn RH, et al. Metagenomic analysis of the microbiota in the highly compartmented hindguts of six wood- or soil-feeding higher termites. Microbiome. 2015;3:56. 26607965 10.1186/s40168-015-0118-1 PMC4660790

[96] Mikaelyan, A., Meuser, K. & Brune, A. Microenvironmental heterogeneity of gut compartments drives bacterial community structure in wood- and humus-feeding higher termites. *FEMS Microbiol. Ecol.* 2017;93:fiw210. 10.1093/femsec/fiw210 27798065

[97] Mikaelyan A, Dietrich C, Köhler T, Poulsen M, Sillam-Dussès D, Brune A. Diet is the primary determinant of bacterial community structure in the guts of higher termites. Mol Ecol. 2015;24:5284–95. 26348261 10.1111/mec.13376

[98] Huang Z, Ni G, Wang F, Zhao X, Chen Y, Zhang L, Qu M. Characterization of a thermostable lichenase from *Bacillus subtilis* B110 and its effects on β-glucan hydrolysis. J Microbiol Biotechnol. 2022;32:484–92. 10.4014/jmb.2111.11017. 34949743 10.4014/jmb.2111.11017 PMC9628817

[99] Kholousi Adab, F., Mehdi Yaghoobi, M. & Gharechahi, J. Enhanced crystalline cellulose degradation by a novel metagenome-derived cellulase enzyme. *Sci Rep*. 2024;14:8560. 10.1038/s41598-024-59256-4. 10.1038/s41598-024-59256-4 PMC11014956 38609443

