# Supplementary material for: Unveiling lignocellulolytic potential: a genomic exploration of bacterial lineages within the termite gut
Source: Microbiome. 2024 Oct 15;12:201. doi: 10.1186/s40168-024-01917-7 (PMC11481507; doi:10.1186/s40168-024-01917-7)
Supplement: Supplementary file 3 — Additional file 2: Figure S1. UMAP biplot of the distribution of CAZymes of termite-associated bacteria color-coded according to phylum in (A) lower termites, (B) higher termites, (C) termite diet, and (D) gut compartment. Figure S2. Distribution of phylogenetic signal across termite-associated bacterial phyla tested against random effects. Figure S3. The drivers of uniqueness in the distribution patterns of (hemi)cellulases and lignin-modification enzymes in 2,223 termite-associated bacterial MAGs. Figure S4. The gene density of cellulases (A) and hemicellulases (B) among the MAGs of Firmicutes, Spirochaetota,Fibrobacterota, and Bacteroidota from lower and higher termites. Figure S5. Biplot of the PCA based on the distribution of lignin-modifying enzymes, color coded according to the phylum to which each genome belongs (A) and according to the gut compartment sampled for the metagenomes (B). Figure S6. Distribution of gene densities in CAZymes with pectinase activities in MAGs from different bacterial phyla. The insets break down the two most abundant phyla, Bacteroidota and Planctomycetota, to the order level. [file 40168_2024_1917_MOESM2_ESM.pdf]

Unveiling Lignocellulolytic Potential: A Genomic Exploration of Bacterial Lineages within the Termite Gut

João Felipe M. Salgado[1], Vincent Hervé[1], Manuel A. G. Vera[1], Gaku Tokuda[3], Andreas Brune[1]*.

Supplementary figures

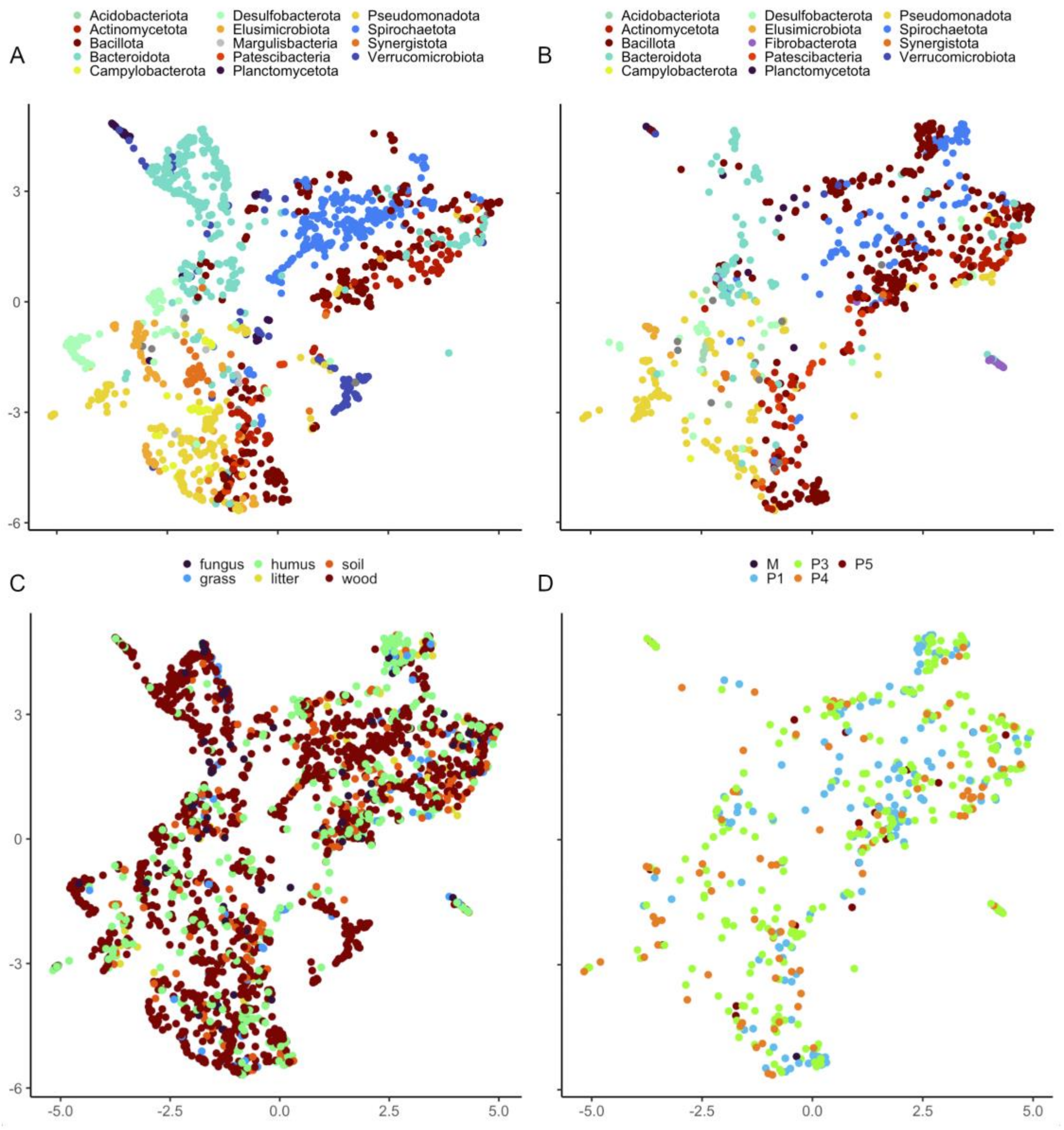

Supplementary Figure 1. UMAP plot (stress=0.12; Component 1 on x-axis and Component 2 on y-axis) of the distribution of CAZymes of termite-associated bacteria color-coded according to phylum in (A) lower termites (R=0.3, R^2=0.31; p=0.001), (B) higher termites (R=0.44, R^2=0.41; p=0.001), (C) termite diet (R=-0.02, R^2=0.001; p>0.5), and (D) gut compartment (R=0.01, R^2=0.04; p>0.5). The theoretical distances were calculated based on the KNN score.

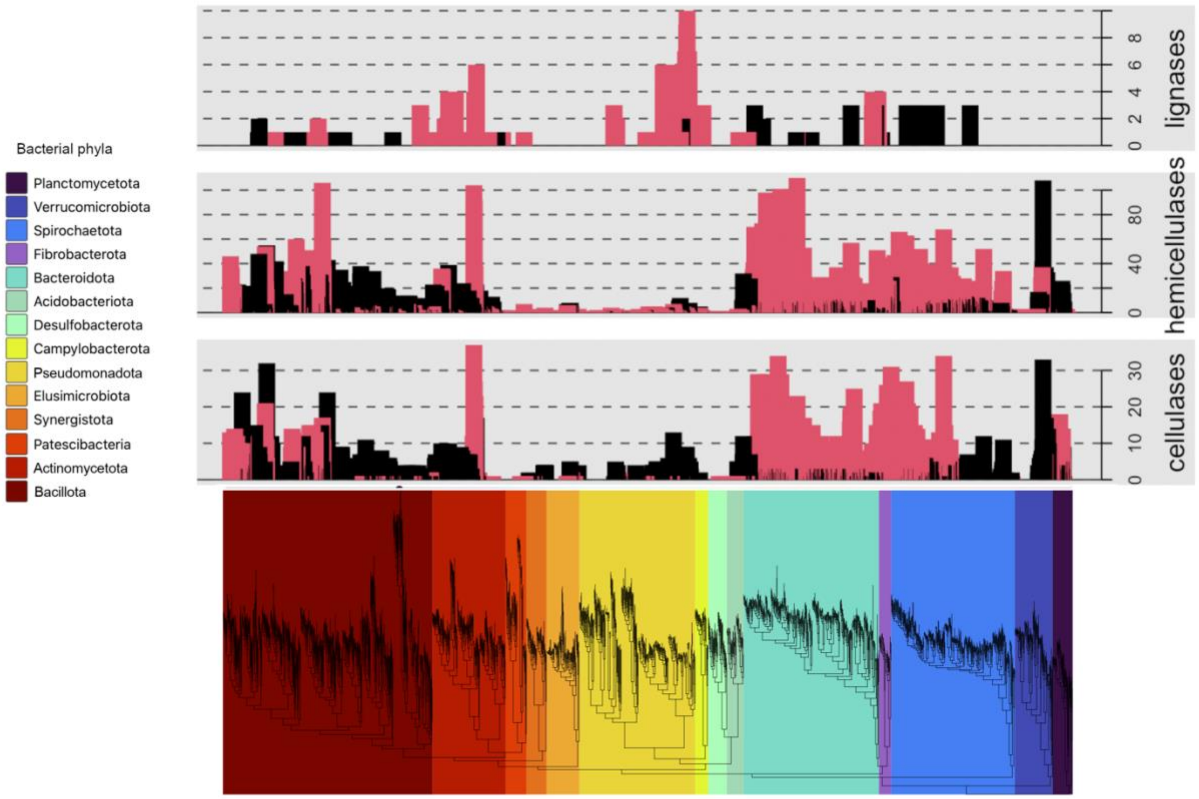

Supplementary Figure 2. Distribution of phylogenetic signal across termite-associated bacterial phyla tested against random effects (λ = 0.004, Cmean = 0.005, p > 0.05). The total number of CAZyme families for each of the activities of interest are given on the y axis. Cellulases and hemicellulases (Global λ = 0.81, Cmean = 0.6, p < 0.001), as well as lignases (Global λ = 0.9, Cmean = 0.5, p < 0.001) across MAGs are mapped to the bacterial phylogeny. Bins whose distributions have significant phylogenetic signal (p < 0.05) are indicated in red.

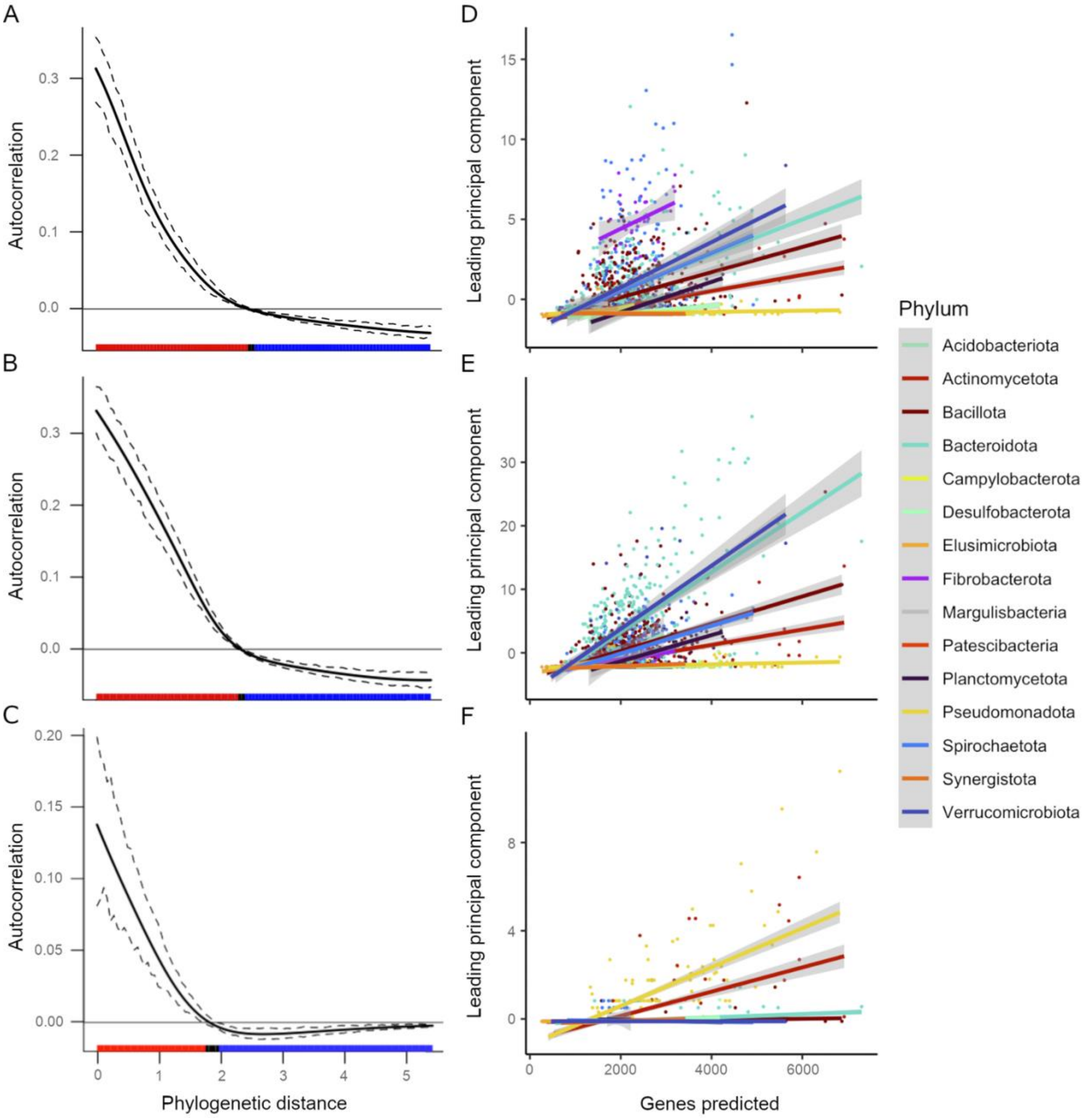

Supplementary Figure 3. The drivers of uniqueness in the distribution patterns of (hemi)cellulases and lignin-modification enzymes in 2,223 termite-associated bacterial MAGs. Phylogenetic correlograms displaying the correlation (y-axis) between the distribution of CAZymes and the phylogenetic distances (x-axis) in (A) cellulases, (B) hemicellulases and (C) lignin-modification enzymes. The solid black line represents Moran's I (I > 0.67, p < 0.001), and the confidence intervals were calculated using 1,000

bootstraps. The horizontal line indicates the value of autocorrelation under null hypothesis. Ranges in red represent positive autocorrelation; ranges in blue represent negative autocorrelation. (D-F) The linear correlation between the per-MAG main explanatory variable (y-axis) and the total amount of predicted genes (x-axis). Explanatory variables are shown as follows: (D) Cellulases; explained variance = approx. 97%, (E) Hemicellulases; explained variance = approx. 75%, and (F) lignin-modifying enzymes; explained variance = approx, 84%. The theoretical distances were calculated based on the eigen decomposition of the variables and are color-coded according to bacterial phyla.

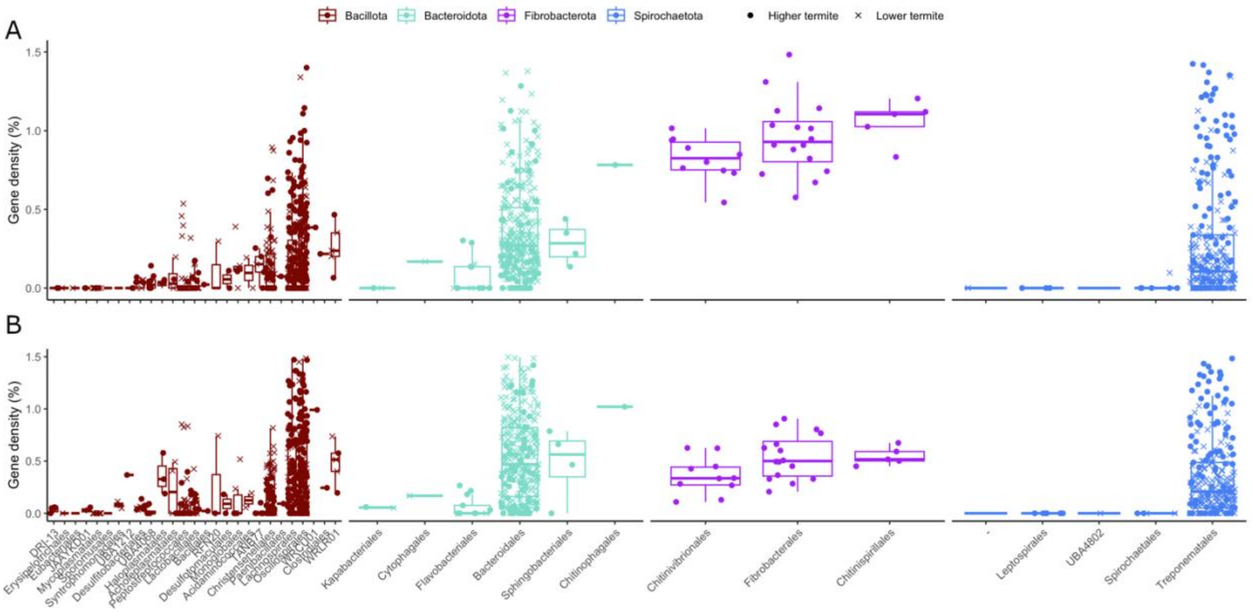

Supplementary Figure 4. The gene density of cellulases (A) and hemicellulases (B) among the MAGs of Firmicutes, Spirochaetota, Fibrobacterota, and Bacteroidota from lower and higher termites. Each datapoint represents the relative abundance (%) of genes assigned to the respective function based on the total number of genes in a MAG.

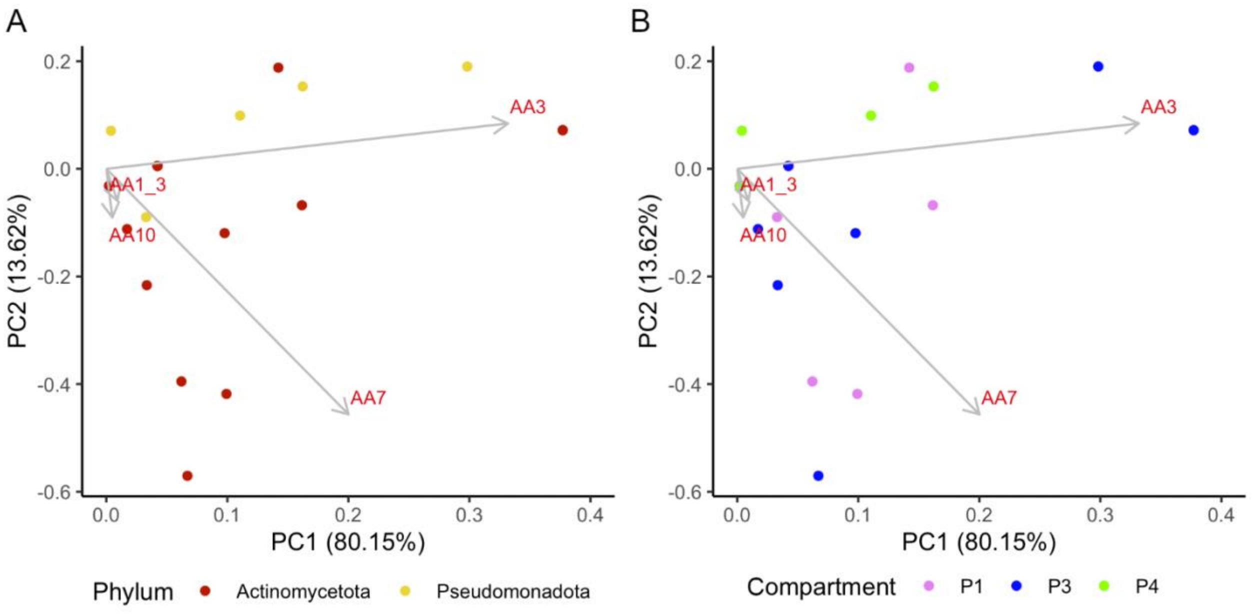

Supplementary Figure 5. Biplot of the PCA based on the distribution of lignin-modifying enzymes, color coded according to the phylum to which each genome belongs (A) and according to the gut compartment sampled for the metagenomes (B).

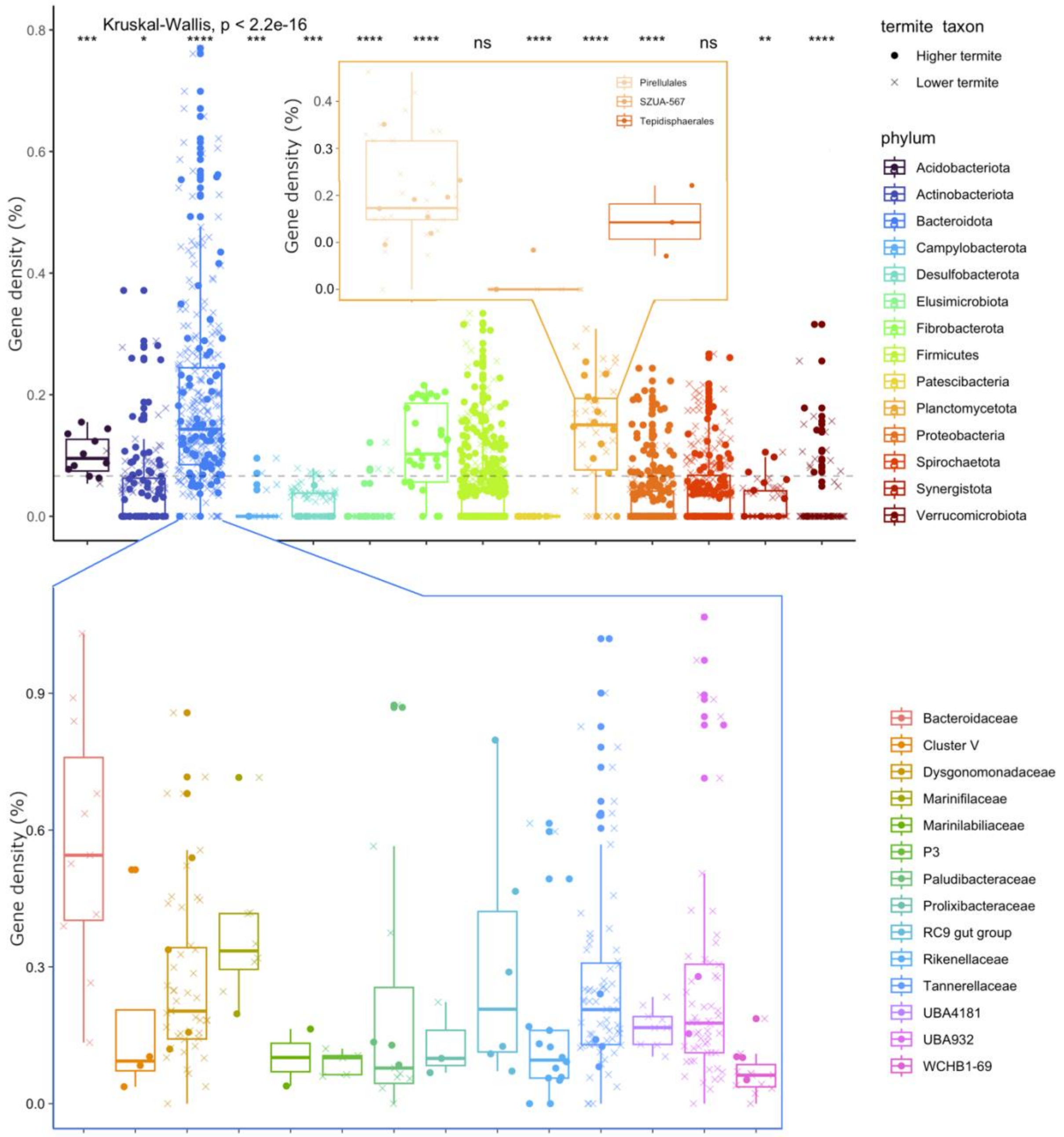

Supplementary Figure 6. Distribution of gene densities in CAZymes with pectinase activities in MAGs from different bacterial phyla. The insets break down the two most abundant phyla, Bacteroidota and Planctomycetota, to the order level.
